# d-Glucose Adsorption on the TiO_2_ Anatase (100) Surface: A Direct Comparison Between Cluster-Based and Periodic Approaches

**DOI:** 10.3389/fchem.2021.716329

**Published:** 2021-08-31

**Authors:** Valeria Butera, Arianna Massaro, Ana B. Muñoz-García, Michele Pavone, Hermann Detz

**Affiliations:** ^1^ CEITEC - Central European Institute of Technology Central European Institute of Technology, Brno University of Technology, Brno, Czech; ^2^ Department of Chemical Sciences, Università di Napoli Federico II, Comp Univ Monte Sant’Angelo, Naples, Italy; ^3^ Department of Physics “Ettore Pancini”, Università di Napoli Federico II, Comp Univ Monte Sant’Angelo, Naples, Italy; ^4^ Center for Micro and Nanostructures and Institute of Solid State Electronics, Vienna, Austria

**Keywords:** titanium dioxide, density functional theory, cluster approach, PBC calculations, glucose adsorption

## Abstract

Titanium dioxide (TiO_2_) has been extensively studied as a suitable material for a wide range of fields including catalysis and sensing. For example, TiO_2_-based nanoparticles are active in the catalytic conversion of glucose into value-added chemicals, while the good biocompatibility of titania allows for its application in innovative biosensing devices for glucose detection. A key process for efficient and selective biosensors and catalysts is the interaction and binding mode between the analyte and the sensor/catalyst surface. The relevant features regard both the molecular recognition event and its effects on the nanoparticle electronic structure. In this work, we address both these features by combining two first-principles methods based on periodic boundary conditions and cluster approaches (CAs). While the former allows for the investigation of extended materials and surfaces, CAs focus only on a local region of the surface but allow for using hybrid functionals with low computational cost, leading to a highly accurate description of electronic properties. Moreover, the CA is suitable for the study of reaction mechanisms and charged systems, which can be cumbersome with PBC. Here, a direct and detailed comparison of the two computational methodologies is applied for the investigation of d-glucose on the TiO_2_ (100) anatase surface. As an alternative to the commonly used PBC calculations, the CA is successfully exploited to characterize the formation of surface and subsurface oxygen vacancies and to determine their decisive role in d-glucose adsorption. The results of such direct comparison allow for the selection of an efficient, finite-size structural model that is suitable for future investigations of biosensor electrocatalytic processes and biomass conversion catalysis.

## Introduction

Titanium dioxide has been the focus of intense research in materials science over the past few decades, thanks to the wide range of technological applications, from biomedicine (Paunesku et al., 2003; Wu et al., 2014) to environment (Kapilashrami et al., 2014; Bella et al., 2017; Bella et al., 2018; Massaro et al., 2020) and photochemistry (Bai et al., 2014; Lettieri et al., 2020). The easy production of TiO_2_-based materials at the nanoscale level is enabled by the high stability they exhibit under extreme conditions. The most common polymorphs are as follows: 1) rutile, which is the most thermodynamically stable bulk phase, 2) anatase, which is found to be more stable at the nanoscale (Zhang and Banfield, 1998; Ranade et al., 2002), and 3) brookite. Nanostructured thin films and nanoarrays with large surface areas typically show unique physico-chemical and electronic properties that are different from the bulk. For example, anatase is the most catalytically active mineral and, therefore, the most interesting one for both research and industrial applications, especially related to heterogeneous photo-(electro)-catalysis (Linsebigler et al., 1995; Guo et al., 2019; Piccolo et al., 2020). TiO_2_ nanoparticles have been found highly effective for the selective catalytic conversion of biomass to value-added products. For example, glucose produced from cellulose through hydrolysis in an aqueous medium can be transformed into useful molecules such as alkyl glucosides, 5-hydroxymethylfurfural, levulinic acid, and gluconic acid (van Putten et al., 2013; Kuo et al., 2014).

On the other hand, the good biocompatibility of TiO_2_-based nanomaterials allowed also for the design and development of innovative sensing devices (Bao et al., 2008; Manera et al., 2008; Wang et al., 2010; Abbasi and Sardroodi, 2018; Wang et al., 2020). General requirements for efficient electrochemical sensors and biosensors are good selectivity, fast response, and reproducible detection of specific chemical and biochemical compounds. Owing to their high sensitivity to glucose, hydrogen peroxide, and cancer cells, TiO_2_-based nanomaterials have been proposed as biosensing materials for the detection of blood glucose in diabetes mellitus patients and early monitoring of cancer (Doong and Shih, 2010; Qiu et al., 2011; Srivastava et al., 2013).

Therefore, the interaction of glucose with all the possible surfaces of anatase nanoparticles is of pivotal importance to design new, more effective nanodevices for sensing along with new efficient and selective catalysts for biomass conversion. The relevant features for both the applications regard the molecular recognition event and its effect on the nanoparticle electronic structure. Here, we address both these features using state-of-the-art first-principles methods, covering two possible modeling approaches. From a general perspective, modern quantum mechanics methods based on density functional theory (DFT) can provide important information on the adsorption processes while elucidating the related physical and chemical properties of the investigated TiO_2_ phase. There are two approaches in the modeling of heterogeneous processes on surfaces: periodic boundary conditions (PBCs) and the cluster approach (CA). The most commonly used PBC approach allows for the investigation of extended surfaces and provides an accurate description of the structural properties of the surface and molecular adsorbates. However, the semilocal functionals usually adopted for this kind of calculations do not account properly for exchange and correlation effects in transition metal oxides, leading to self-interaction errors and, therefore, inaccurately describe the electronic band gaps of non-metallic materials. In general, more accurate exchange-correlation functionals are computationally expensive when used in PBC calculations. The DFT + U method (Dudarev et al., 1998) is the most suitable alternative. This approach takes into account the on-site Coulombic repulsion among localized d-electrons by incorporating an extra energetic penalty for delocalization at a relatively computationally low cost. Calculations of charged systems in PBCs are problematic owing to spurious interactions between the charges in different periodic images that can affect the physical picture. Furthermore, due to the highly demanding computational cost, they are not suitable for the investigation of complex reaction mechanisms involving several stationary points. On the other hand, the CA allows using hybrid functionals with low computational cost, leading to a highly accurate description of electronic properties. The ability to add/subtract a charge carrier without suffering from interactions with similar charge carriers due to PBC (Butera and Caspary Toroker, 2016) makes CAs suitable for the study of charged systems. Thanks to the reduced size, the CA can be used to intercept all the stationary points, including the more complex transition states, involved in the catalytic cycle, and to determine the rate-determining states (D’Arienzo et al., 2017; Butera et al., 2018; Butera et al., 2021). However, the selection of the cluster models is not trivial. Particular attention needs to be paid to the choice of the cluster size as a perfect balance between accuracy and computational cost (Keith et al., 2015), and the most suitable procedures to saturate the peripheral oxygen atoms (Butera and Toroker, 2017). Moreover, the accuracy of cluster model approaches’ results largely depends on the localized nature of the interaction between the surface and adsorbates. From this perspective, the combination of both PBC and CAs can be useful to achieve an in-depth understanding of the structureproperty relationship and can represent a promising strategy for the design of novel TiO_2_ anatase-based sensors and catalysts.

In particular, we propose a DFT-based investigation of molecular adsorption of d-glucose on the TiO_2_ (100) anatase surface using the joint cluster and PBC approaches. d-Glucose is the most common building block in the photocatalytic reforming of biomass to hydrogen and carbon dioxide in the presence of titania (Abrahams et al., 1983). Several works have addressed the adsorption of small molecules, such as CO, CO_2_, H_2_, and H_2_O, on the (101) surface (Onal et al., 2006; Chang et al., 2008; Islam et al., 2011; Mino et al., 2014; Martinez-Casado et al., 2018), but the investigation of bigger compounds still represents a great challenge from the computational point of view. The structural model becomes even more demanding when multiple adsorption modes should be considered, as in the case of d-glucose. As a matter of fact, a conclusive understanding of the interaction of glucose with all the TiO_2_ anatase surfaces is still missing. Balducci (2010) has recently investigated the adsorption of glucose on the (101) anatase surface slab in “end-on” and “bridge” adsorption modes via single and two hydroxyl groups, respectively, in both molecular and dissociative coordination. The results reported in his work underline that the glucose molecule prefers an orientation normal to the titania surface, but the dihedral angle can vary depending on the constraints imposed by the particular hydroxyl group(s) involved in the adsorption. The binding is favorable in all cases, with molecular adsorption being preferred over dissociative one. However, this work does not cover all the other relevant surfaces of anatase nanoparticles as, for example, the (100) or the high energy (001) ones.

Several studies can be found in the recent literature focusing on the role of different anatase surfaces in determining the activity toward a specific application (Lundqvist et al., 2006; De Angelis et al., 2014). The (101) anatase surface termination has shown promising performance in photo-driven charge transfer systems (Maity et al., 2018). Yu et al. (2014) suggested that the (101) facet should be the reactive surface in photocatalytic reactions because it has a lower conduction band edge. Triggiani et al. (2015) have pointed out that the adsorption of trimethylamine (TMA) on the (100) facet affects the overall electronic structure with newly occupied states in the TiO_2_ anatase band gap, leading to new principles for the design of hybrid organic-inorganic systems composed by amine-based molecular moieties exposed on titania nanorods. Even though most adsorption studies are dedicated to the (101) surface as it represents the most thermodynamically stable lattice termination (Lazzeri et al., 2001), Triggiani et al. (2015) underline the importance of considering different TiO_2_ facets. Based on these considerations, the TiO_2_ (100) anatase surface has been selected in this work. Following the work of Balducci (2010), we do not consider the dissociative mechanism in this work, which has also the aim of a neat comparison between the cluster and PBC schemes in order to select the best choice for following extensive studies on this system.

## Materials and Methods

### Cluster Approach

**FIGURE 1:**
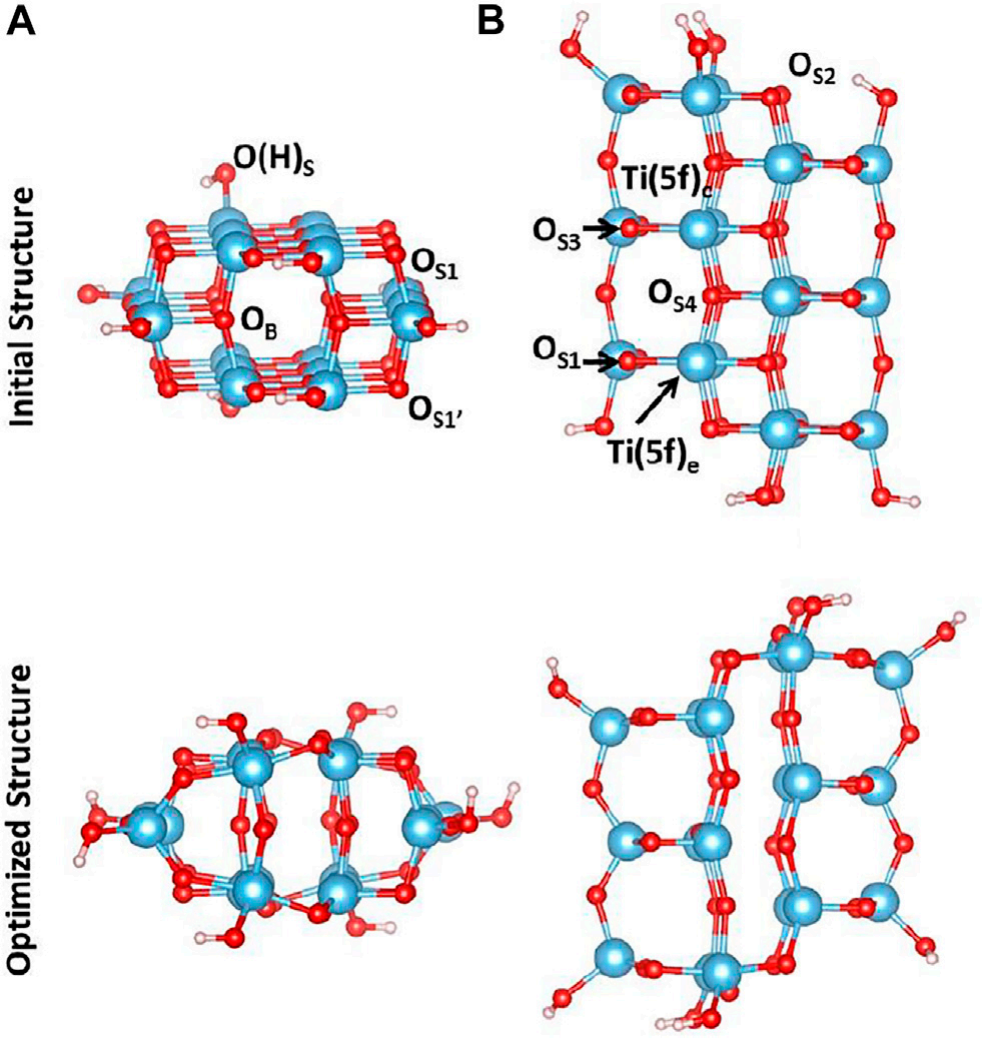
**(A)** Side and **(B)** top view of initial **(top)** and optimized **(bottom)** structures at the B3LYP level of theory of the cluster model containing 68 atoms. Color code: O, red; H, white; Ti, blue.

The cluster model used in this study has been carved out from the crystallographic structure of anatase. We have cleaved the (100) surface and selected a cluster model that contains 68 atoms, whose initial and optimized structures are shown in Figure 1. During the optimization, all the atoms have been allowed to relax. Following a very common procedure, OH groups have been added along the Ti–O bond vectors, replacing original O atoms present in the crystal structure in order to preserve the symmetry. Frequency calculations in the harmonic approximation have been performed to confirm that each cluster geometry represents a minimum in the potential energy surface.

All the cluster calculations have been performed using the open-source ORCA software (Neese, 2012; Neese, 2017). The well-known B3LYP (Foster and Weinhold, 1980) functional has been first selected. O, H, and C atoms are described by the full electron 6–31G* basis set, while the SDD (Andrae et al., 1990) pseudo potential is used for Ti metal atoms. As it will be discussed in the next section, all the calculations have also been performed introducing the BeckeJohnson (BJ) damping dispersion corrections, D3BJ, as implemented in the ORCA software. Further calculations with PBE0 (Perdew et al., 1996; Perdew et al., 1997; Adamo and Barone, 1999) functional have been performed and are discussed in detail in the Supporting Information.

### Periodic Boundary Condition Approach

**FIGURE 2:**
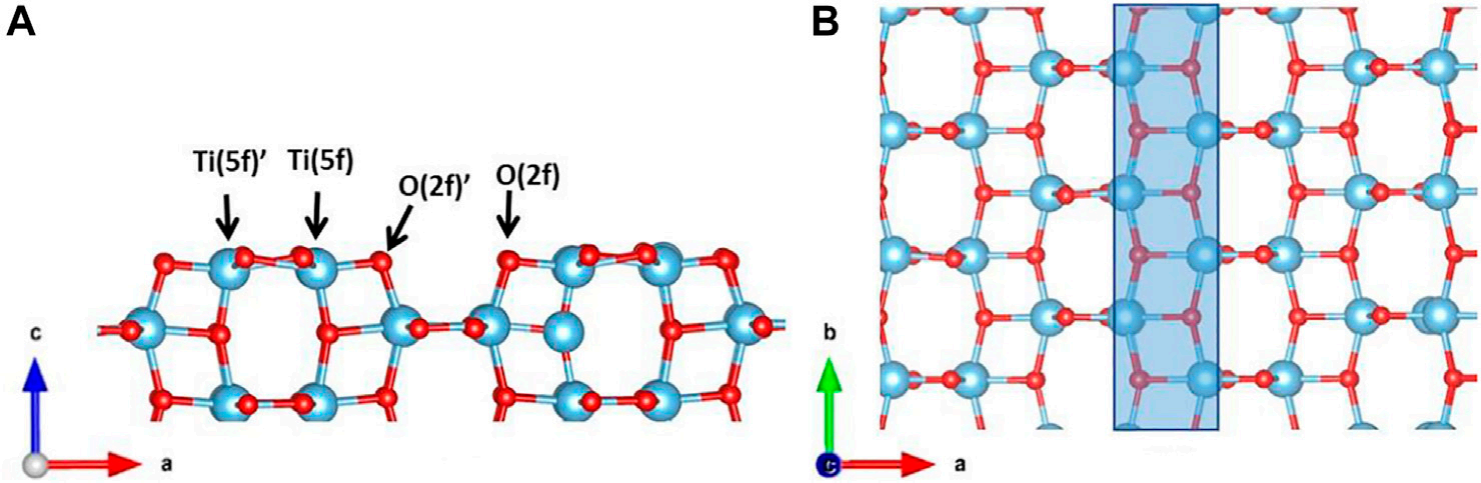
**(A)** Side and **(B)** top view of the TiO_2_ anatase (100) surface slab model. The highlighted area in blue indicates the gap between two rows of building blocks. Color code: O, red; H, white; Ti, blue.

Spin-polarized DFT calculations have been performed within PBC using the PBE (Perdew et al., 1996; Perdew et al., 1997; Adamo and Barone, 1999) exchange-correlation functional based on the generalized gradient approximation and the projected augmented wave pseudopotential to treat core electrons, as implemented in the VASP code [version 5.3.4 (Kresse and Furthmüller, 1996)]. We adopt the on-site Coulomb correction, U, for the Ti 5d states (DFT + U approach) (Dudarev et al., 1998) with a value of U = 3.3 eV (Finazzi et al., 2008) in order to overcome the self-interaction error (SIE) occurring with highly localized electrons and the DFT-D3 scheme developed by Grimme (2006) with the BJ damping function (Grimme et al., 2010; Grimme et al., 2011) to take into account vdW forces at the adsorbatesurface interface. We select a cutoff energy of 500 eV and a 4 × 4 × 2 k-points sampling mesh based on the Monkhorst-Pack scheme to converge the plane-wave basis set. Optimized lattice parameters of the TiO_2_ anatase tetragonal unit cell are *a* = 3.828Å and *c* = 9.800Å, which are in good agreement with the experimental values (*a* = 3.782Å and *c* = 9.502Å) (Burdett et al., 1987). We build up the structural model for the TiO_2_ anatase (100) surface in order to investigate the glucose adsorption. The slab model contains 216 atoms (72 f.u.) and a vacuum layer of ∼20Å along the c direction (Figure 2) required to place the glucose molecule while avoiding image interaction. The final dimensions of the supercell are a = 19.620Å, b = 15.305Å, and c = 25.000Å. The threshold for energy convergence is set to 10^–4^ eV. For the slab optimization, only the atoms in the first layer and from the glucose molecule are allowed to relax until the forces are below 0.03 eV/Å. Because of the large dimension, the Brillouin zone for the slab model has been sampled only in the Γ point.

## Results and Discussion

### d-Glucose Adsorption

The optimization of the selected cluster model at the B3LYP level of theory using SDD ECP for Ti and 6–31G* basis sets for O and H shows good agreement with previously reported data. Our computed Ti-O_av_ distance (2.02 Å) matches the experimental (1.95 Å) and theoretical (1.86–1.91 Å) values reported in Balducci (2010), confirming the reliability of the selected computational protocol for the geometry optimizations, with no need to apply larger basis sets that would have made the computation too demanding.

As shown in Figure 1, upon geometry relaxation, the cluster undergoes a structural rearrangement leading to a partial distortion of the TiO_2_ lattice. More significant changes involve the oxygen atoms at the edges of the cluster model: the distance between O_S1_ and O_S1’_ shorten from 3.815Å to 2.837Å and, as a consequence, the O_S1_-Ti-O_S1’_ angle changes from 150.8° to 104.8°. On the other hand, the distance between O_S2_ and O_S2’_ increases from 4.107Å to 4.793Å. Thus, the lattice distortion observed for this cluster consists of a compression along the z direction and an in-plane elongation.

**FIGURE 3:**
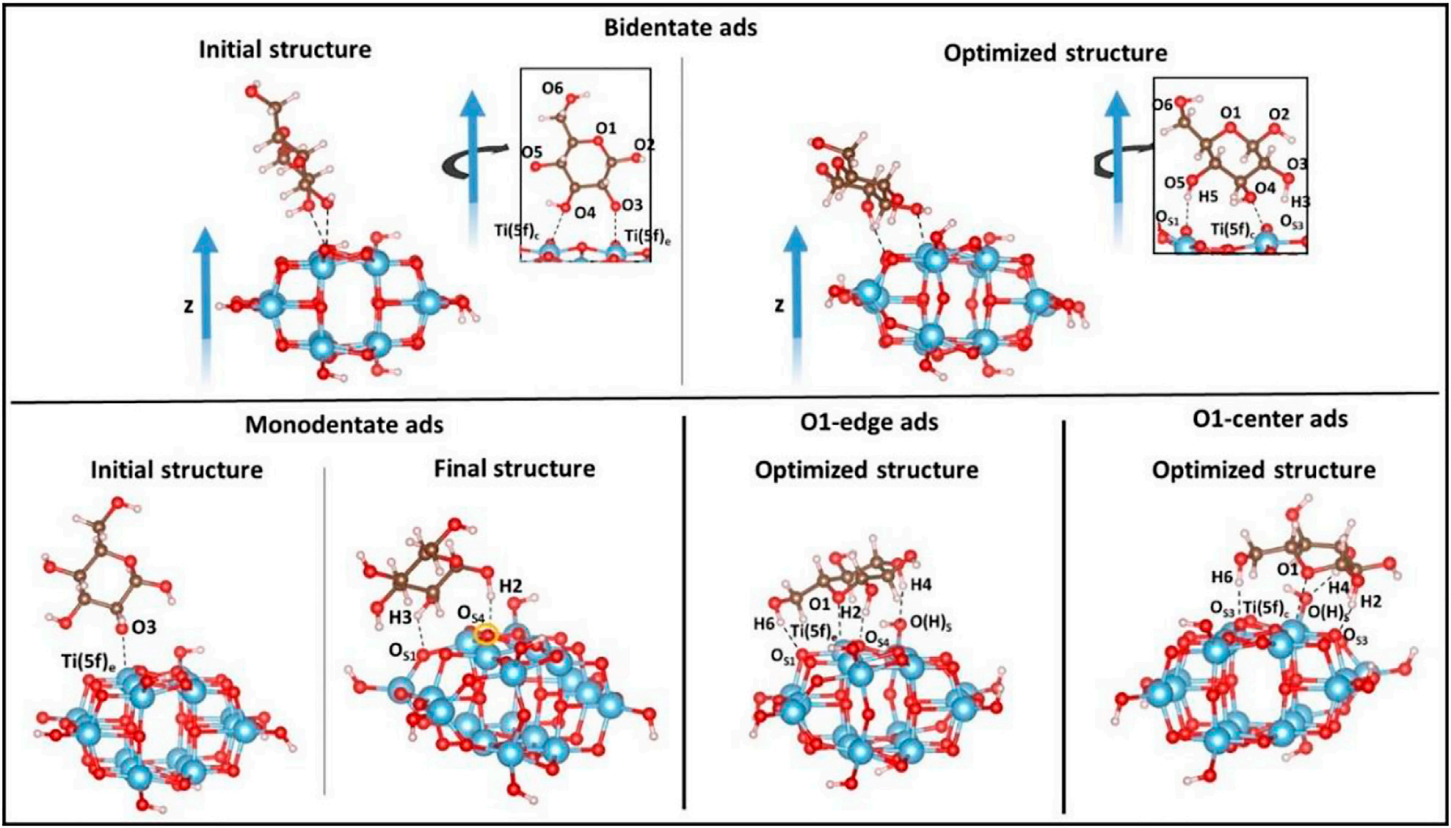
Different adsorption modes of d-glucose on the TiO_2_ anatase (100) cluster surface investigated in this work. Color code: O, red; H, white; Ti, blue; C, brown.

Once the cluster model has been optimized, we investigate the molecular adsorption of d-Glucose on different sites of the TiO_2_ surface in order to identify the more favorable mode. d-Glucose contains five different OH groups and one ether O atom (see Figure 3 for the corresponding labeling that we are going to use throughout the text). Since the systematic investigation of all the possible sites would be highly demanding, we choose four different adsorption modes as follows: 1) bidentate adsorption through two adjacent OH groups, that is*.*, O3 and O4; 2) monodentate adsorption via one OH group, that is, O3; 3) adsorption involving the ether oxygen atom, O1, on a Ti atom of anatase located at the cluster edge, Ti (5f)_e_, (O1-edge ads), and 4) on the center, Ti (5f)_c_, (O1-center ads). The relative adsorption energies, *E(cluster)*_*ads*_, are calculated as:

$$E{\left(cluster\right)}_{ads}=E_{cluster+D-glucose}-\;E_{cluster}-E_{D-glucose},$$ (1)

where *E*_*cluster*_ is the energy of the optimized cluster, *E*_*D-glucose*_ is the energy of the d-glucose molecule, and *E*_*cluster+D-glucose*_ is the energy of the cluster when the d-glucose molecule is coordinated to the surface. Figure 3 shows the optimized structures for each selected adsorption mode. For bidentate and monodentate adsorption modes, the initial structures are also shown in order to underline the rotation of the sugar molecule upon relaxation, while in the case of O1-edge and O1-center, the rearrangement of glucose can be neglected.

As mentioned above, in the bidentate coordination, the d-glucose molecule rotates upon geometry optimization since some stabilizing interactions are established at the interface: two H-bonds (H3---O_S3_, 1.678Å and H5---O_S1_, 1.755Å) involving OH groups from the sugar and O atoms located at the edges of TiO_2_ surface; one Ti-O bond (2.343Å) involving the Ti atom located at the center, Ti (5f)_c_, and the nearby OH group from the sugar molecule, O4. In the monodentate coordination, the sugar molecule is initially coordinated via the O3-Ti (5f)_e_, but a partial rotation occurring during the optimization leads to the breaking of the Ti-O bond and to the formation of two H-bonds (H2---O_S4_, 1.712Å and H3---O_S1_, 1.708Å), thus showing that the monodentate coordination actually does not take place. In the O1-edge coordination, a Ti-O bond (2.474Å) is established between the O ether atom of the glucose and the Ti (5f)_e_ atom located at the edge of the cluster surface, and also three H-bonds are formed between an OH group from the glucose and an O atom from the TiO_2_ surface (H6---O_S1_, 1.750Å, H2---O_S4_, 1.559Å and H4---O(H)_S_, 1.770Å), with the third one involving the terminal oxygen added to saturate the cluster after its cleavage. A very similar adsorption is found when the O1-center ads coordination is considered, with the formation of 3 H-bonds (H2---O_S3_, 1.520Å; H6---O_S3_, 1.814Å; H3---O(H)_S_, 1.747Å) and a Ti-O bond distance (Ti (5f)_c_-O1, 2.645Å). As a consequence of the glucose adsorption, all the bonds involving the atoms of the cluster surface interacting with the sugar molecule groups undergo elongation. The adsorption energies are calculated according to Eq. 1, and the results are summarized in Table 1. The overall negative values show that all the adsorption processes are exothermic, with the O1-center ads being the most favored adsorption mode (for which B3LYP-D3BJ energy is also reported in the table).

**TABLE 1:** Adsorption energies of d-glucose, E (cluster)_ads_, on four different sites at the B3LYP level of theory. For the most favored O1-center mode, the B3LYP-D3BJ energy is also reported. Energies are given in eV.

	Bidentate	Monodentate	O1-edge	O1-center	
				B3LYP	B3LYP-D3BJ
**E (cluster)**_**ads**_	−2.17	−2.09	−2.23	−2.49	−3.01

To the best of our knowledge, the investigation by the cluster model approach of the adsorption of d-glucose on anatase has not been reported in the literature. Lundqvist et al. (2006) have reported a DFT investigation of pyridine phosphonic and carboxylic acids via a monodentate binding mode on the (101) surface of the (TiO_2_)_46_ anatase cluster. They found that all the adsorbates bind to a Ti (5f) surface atom, which consequently achieves a six-fold coordination, and also underline the presence of Ti=O double bonds that participate in the adsorption process of both pyridine phosphonic and carboxylic acids via the formation of H-bonds with their OH groups. Both the adsorptions are exothermic, with calculated energies of −3.56 and −1.82 eV for pyridine phosphonic and carboxylic acids, respectively. Our results do not show the formation of any Ti=O bond on the anatase surface and suggest that the monodentate coordination mode is not the favored one. Furthermore, according to our results, Ti (5f) is not the active atom in the adsorption process. However, we do believe that those differences are due to the diverse studied surface facets and adsorbates.

Bandura et al. (2004) have studied the adsorption of a water molecule on the anatase via an embedded cluster model. The authors report that the associative mechanism is more favorable than the dissociative one and in better agreement with the experimental results. They also found that both associative and dissociative adsorption structures show no presence of H-bond interactions between the water molecule and the surface. Conversely, our results have underlined the importance of hydrogen bond formation in the adsorption modes, in agreement with Lunell’s findings (Lundqvist et al., 2006) discussed above. Associative adsorption on (101) and (100) has been reported as the favored mechanism also by Selloni and co-workers (2010) and Triggiani et al. (2015). Supported by those results, we decided not to consider d-glucose dissociative adsorption at this stage of our study.

**FIGURE 4:**
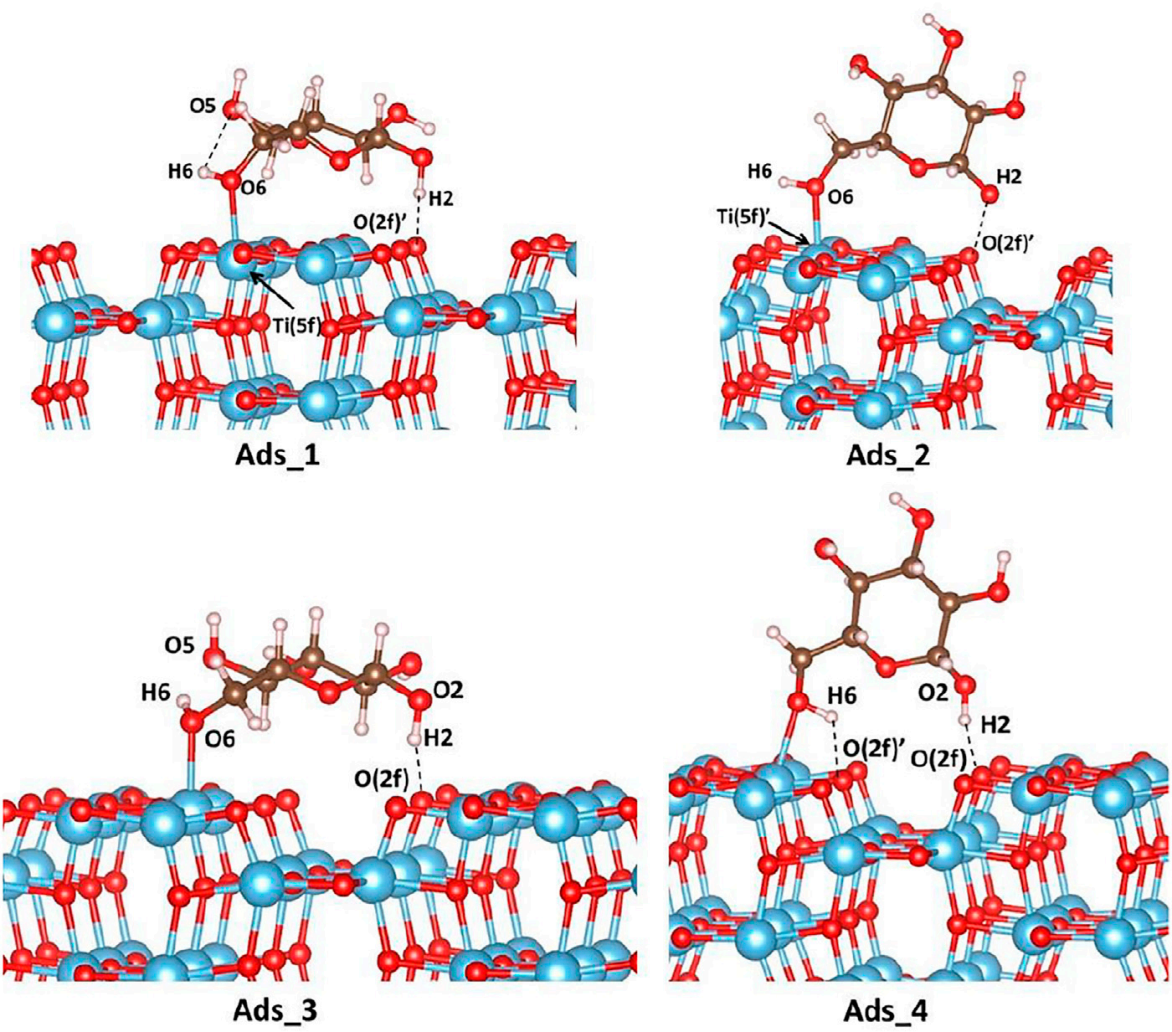
Adsorption modes of d-glucose on the (100) TiO_2_ anatase surface. Color code: O, red; H, white; Ti, blue; C, brown.

As mentioned above, the adsorption of d-glucose on anatase (100) has been investigated also by PBC calculations. In a similar way to cluster calculations, the adsorption energy of glucose on the stoichiometric surfaces is calculated as follows:

$$E{\left(PBC\right)}_{ads}=E_{surface+D-glucose}-\;E_{surface}-E_{D-glucose},$$ (2)

where $E_{surface+D-glucose}$ is the total energy of the molecule adsorbed on the surface, $E_{surface}$ is the total energy of the optimized surface, and $E_{D-glucose}$ is the total energy of the molecule. Four different adsorption sites have been investigated as shown in Figure 4, and the corresponding adsorption energies are listed in Table 2. In the case of Ads_1 and Ads_2, the d-glucose molecule is located on the terrace area of the TiO_2_ surface and can therefore be directly compared to the cluster models. On the other hand, in Ads_3 and Ads_4, the sugar molecule is located on the gap between two rows, so we select another cluster model to simulate this adsorption site and discuss the related results in the Supporting Information.

**TABLE 2:** Adsorption energies, E (PBC)_ads_, computed according to Eq. 2 at PBE and PBE-D3BJ levels of theory for each explored binding site. Values are given in eV.

	Ads_1	Ads_2	Ads_3	Ads_4
**PBE**	−0.63	−0.33	−0.62	−0.60
**PBE-D3BJ**	−1.51	−0.90	−1.42	−1.10

As shown in Table 2, for all four investigated cases, much stronger adsorption energies are calculated when dispersion corrections are taken into account and, therefore, only those will be discussed below. Ads_1 is found to be the most favored adsorption mode. The sugar molecule, located on the flat area of the anatase surface, establishes two interactions with TiO_2_: a hydrogen bond of 1.700Å between H2 and O (2f) which is also observed in the cluster calculations with a distance of 1.714Å; a Ti-O bond of 2.248Å between O6 of the glucose molecule and the five-fold coordinated Ti atom, Ti (5f)’, that is not detected in the cluster model. The vertical adsorption described in Ads_2 leads to the same kind of interactions observed in Ads_1. However, the corresponding distances are longer (2.282Å and 2.263Å for O6-Ti (5f)’ and H2---O (2f)’, respectively), which explains the weaker adsorption (less negative adsorption energies). It is noteworthy that from the cluster investigation, vertical adsorption is not predicted, as the d-glucose molecule undergoes spontaneous rotation during the optimization to reach the horizontal orientation (see initial structures in Figure 3). As mentioned above, in Ads_3 and Ads_4 adsorption modes, the sugar molecule is located above the gap. In Ads_3, one hydrogen bond is established between H2 of a sugar molecule and the two-fold coordinated oxygen atom, O (2f), of anatase with a distance of 1.757Å. Another interaction with a distance of 2.238Å involves one of the five-fold coordinated surface titanium, Ti (5f), with the O6 of d-glucose. In Ads_4 mode, the same interaction occurs, that is 1.664Å for O (2f)---H2 and 1.859Å for Ti (5f)-O6, and an extra hydrogen bond of 1.761Å is established between H6 and O (2f).

### BSSE

Discrepancies between the two computational approaches in computing adsorption energies are often found in the literature (Bandura et al., 2004). A possible reason is related to the basis set superposition error (BSSE) arising from the use of the localized atomic orbital scheme in cluster calculations. As a consequence, in the description of the supersystem (TiO_2_ surface with adsorbed d-glucose), the basis set of the supersystem is larger than that used for the component subsystems (TiO_2_ surface and d-glucose molecule separately), thus obtaining an energy that is biased toward the dimer formation due to basis set effects. In order to correct for the BSSE, the counterpoise (CP) method has been used following the Boys and Bernardi formula adapted for our TiO_2_-d-glucose system as implemented in the ORCA software:

$$\Delta E\;\left(CP\right)=E_{AB}^{AB}\left(AB\right)-E_{A}^{A}\left(A\right)-E_{B}^{B}\left(B\right)-[E_{A}^{AB}\left(AB\right)-E_{A}^{AB}\left(A\right)+E_{B}^{AB}\left(AB\right)-E_{B}^{AB}\left(B\right),$$ (3)

where $E_{X}^{Y}\left(Z\right)$ is the energy of fragment X calculated at the optimized geometry of fragment Y with the basis set of the fragment Z. The CP corrections have been calculated referring to the most favored O1-center ads mode at both B3LYP and B3LYP/D3 level of theory. The corrected adsorption energies are reported in Table 3 (uncorrected values are also shown for a direct comparison).

**TABLE 3:** CP corrections for the most favored O1-center adsorption mode at both B3LYP and B3LYP-D3BJ level of theory. Energies are given in eV.

	∆E (CP)		∆E	
	B3LYP	B3LYP-D3BJ	B3LYP	B3LYP-D3BJ
**O1-center**	−1.57	−1.85	−2.49	−3.01

Those results underline that the effect of the BSSE is a significant fraction of the interaction energy, and it should therefore be taken into account. Furthermore, the corrected adsorption energies are qualitatively similar to that calculated with PBC: -1.51 eV for the Ads_1 is comparable to O1-center, in which the d-glucose molecule is located on the flat area of the anatase surface in both PBC and CAs, respectively. Little differences found here can be related to the difference in symmetry and boundary conditions: the absence of PBC in the cluster models may lead to artificial rigidity of the Ti-O bonds demonstrating the larger reactivity of the cluster surface compared with the periodic one.

### Single Contributions to Adsorption Energy

The total adsorption energy, $E_{ads}$, can be decomposed into two main contributions, that is, interaction and distortion energies:

$$E_{ads}=E_{INT}+\;E_{DIST}.$$ (4)

The interaction energy, $E_{INT}$, is given by the difference between the energy of the whole system and that of the single components taken at the final geometry of the adsorbed state, thus accounting for the bare electronic effects occurring at the adsorbatesurface interface:

$$E_{INT}=E_{SYSTEM}-\left(E_{adsorbate}^{*}+E_{surface}^{*}\right)$$ (5)

Which can be further divided into:

$$E_{INT}=E_{INT\left(DISP\right)}+E_{INT\left(no-DISP\right)}\;,$$ (6)

where the values of $E_{INT\left(DISP\right)}$ and $E_{INT\left(no-DISP\right)}$ are taken from the output files of the calculations. The distortion energy, $E_{DIST}$, is given by Eq. 7:

$$E_{DIST}=E_{adsorbate}^{*}+E_{surface}^{*}-E_{adsorbate}-E_{surface},$$ (7)

where the terms with and without the * are taken, respectively, at the geometry of the final adsorbed state and the unperturbed equilibrium structures of every single component. This contribution accounts for the structural arrangements of both molecule and surface occurring upon adsorption.

Such contributions have been calculated for both cluster and PBC calculations referring to the adsorption of d-glucose molecule on the terrace area and parallel to the TiO_2_ surface (O1-center and Ads_1 adsorption modes). The results considering both the approaches are reported in Table 4. Both PBC and CAs show a positive contribution of 0.53 and 1.99eV, respectively, due to the distortion of the anatase surface as a consequence of d-glucose adsorption. However, the very negative terms of the interaction energy indicate a strong interaction between the sugar molecule and the surface favoring the overall adsorption process. Furthermore, our calculations show that the main contribution to the interaction energy is the non-dispersion forces in both PBC and cluster calculations.

**TABLE 4:** Total adsorption energy, E_ads_, and main contributions, E_DIST_, E_INT_, E_INT(DISP)_, E_INT(no-DISP)_, as defined in the text calculated for Ads_1 and O1-center adsorption modes. Energies are given in eV.

	E_ads_	E_Dist_	E_INT_	E_INT (DISP)_	E_INT (no-DISP)_
**Ads_1**	−1.51	0.53	−2.04	−0.87	−1.17
**O1-center**	−3.01	1.94	−4.95	−1.35	−3.60

### Oxygen Vacancy Formation

**FIGURE 5:**
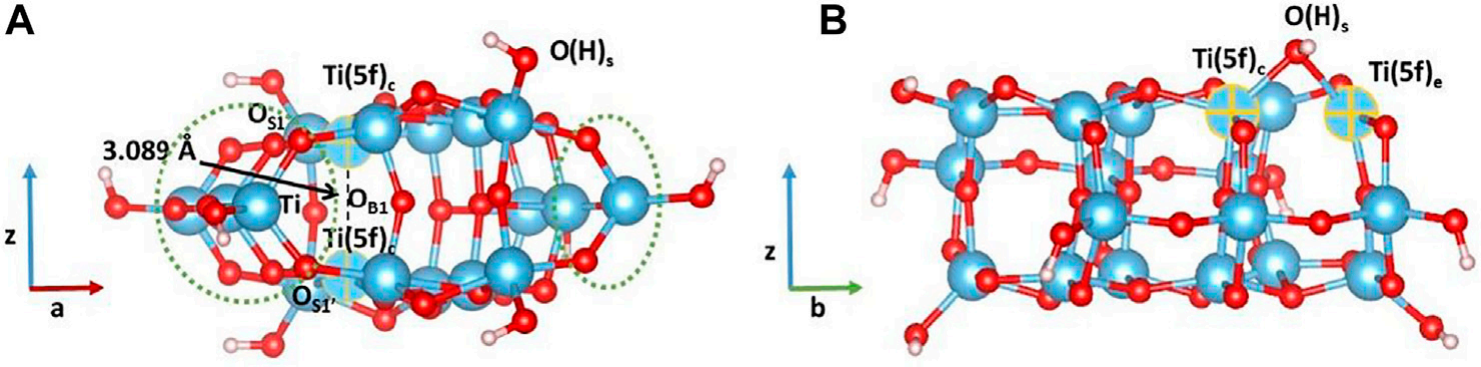
Minimum-energy structures obtained at the B3LYP level of theory for defective cluster models containing one oxygen vacancy at **(A)** subsurface and **(B)** surface. Color code: O, red; H, white; Ti, blue.

In order to provide a realistic view for the interpretation of experimental results, we have also studied point defects like oxygen vacancies. Figure 5 shows the optimized cluster structures containing one subsurface and surface oxygen vacancy, while Table 5 reports the relative vacancy formation energies calculated as:

$$E{\left(V_{O}\right)}_{form}=E{\left(V_{O}\right)}_{cluster}+\;\frac{1}{2}E\left(O_{2}\right)-E\left(cluster\right),$$ (8)

where $E{\left(V_{O}\right)}_{cluster}$ is the energy of the optimized cluster structure with one subsurface/surface oxygen vacancy, $E\left(O_{2}\right)$ is the energy of the oxygen molecule, and E(cluster) is the energy of the optimized cluster structure.

**TABLE 5:** Oxygen vacancy formation energies in both subsurface, $\text{E}{\left({\text{V}}_{\text{O}}\right)}_{\text{form}}^{\text{sub}}$, and surface, $\text{E}{\left({\text{V}}_{\text{O}}\right)}_{\text{form}}^{\text{surf}}$, calculated at B3LYP and B3LYP-D3BJ level of the theory according to Eq. 8. Values are given in eV. Oxygen vacancy formation energies from Ref. 33.

	B3LYP	B3LYP-D3BJ	(101)* 3layers	(001)* 4 layers
$E{(V_{O})}_{\mathrm{form}}^{\mathrm{sub}}$	4.96	5.18	4.25	4.72
$E{\left(V_{O}\right)}_{\mathrm{form}}^{\mathrm{surf}}$	6.04	6.15	4.03	5.08

Our results show that the formation of an oxygen vacancy in the subsurface is easier than on the surface. The two optimized structures are also very different: in the former case, the distance between the two Ti (5f)_c_ showed in Figure 5A decreased from 3.789Å to 3.089Å, while the O_S1_-Ti-O_S1’_ angles underlined in yellow in Figure 5A change from ∼150° to 104°; in the latter, the terminal OH group becomes a bridging moiety with distances being 2.100Å and 2.075Å for Ti (5f)_c_–OH and Ti (5f)_e_–OH, respectively, with no significant angles changes (see Figure 5B). To the best of our knowledge, DFT investigations of the vacancy formation energy on the (100) surface using a cluster model approach have not been reported. Cheng and Selloni (2009) have investigated (101) and (001) defective surfaces using periodic boundary condition calculations considering two different slab sizes, and they have determined the energetics of oxygen vacancies formation at different surface and subsurface sites. In agreement with our cluster results, the authors reported that O vacancies have lower formation energy in the subsurface than at the surface, as shown in Table 2 (n.b. In Table 2, we report only the oxygen vacancy formation energies related to the sites that can be directly compared to those considered in our cluster models). The authors pointed out that the structural relaxations around the vacancy sites show much larger atomic displacements in the subsurface region than at the surface, while the “rigidity” of the surface enhances its stability but at the same time also leads to a very high-energy cost for creating a defect. These results are also confirmed by our cluster calculations, which show a higher degree of distortion of the cluster containing a subsurface than that with a surface defect. The agreement between the two computational approaches underlines that cluster models of medium-size can be used as an alternative less-demanding computational method for the investigation of defect formations.

**FIGURE 6:**
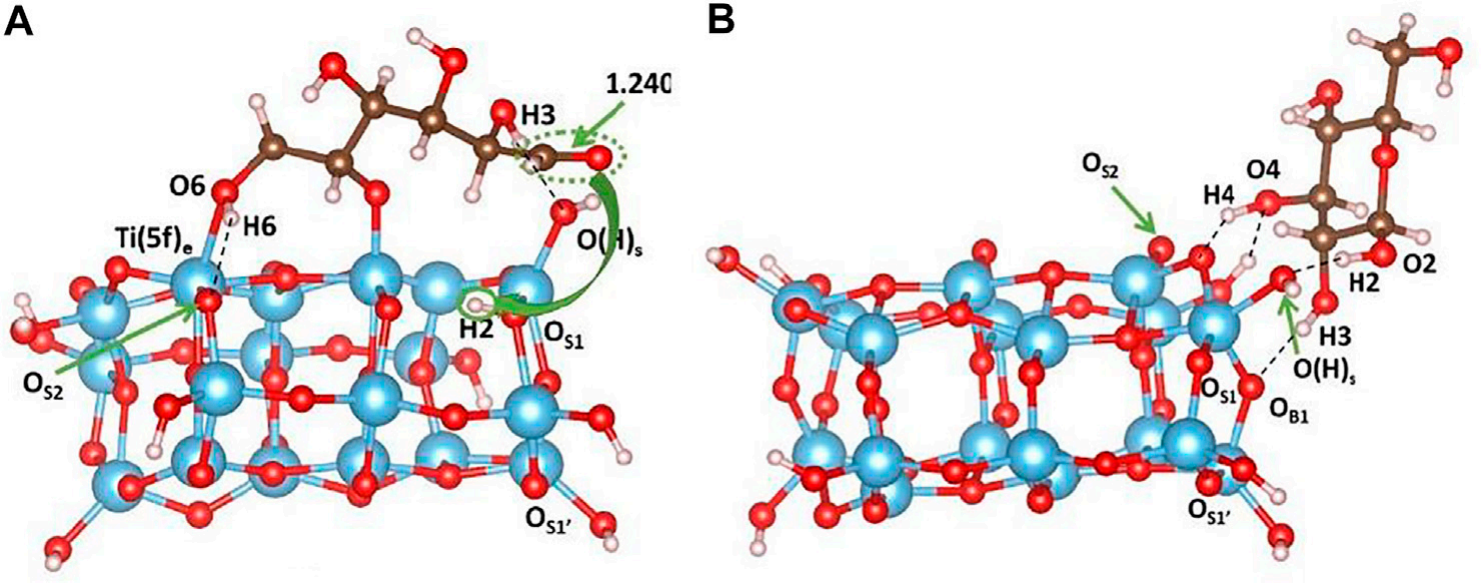
Minimum-energy structures obtained at the B3LYP level of theory for d-glucose adsorption on the cluster containing **(A)** surface and **(B)** subsurface oxygen vacancy. In panel **(A)**, the yellow arrow highlights the H2 transfer from O2 to O_S1_. Color code: O, red; H, white; Ti, blue; C, brown.

We have also investigated and compared the effects of surface and subsurface O-vacancies on the adsorption of sugar molecules. The presence of an O-vacancy in the surface is shown to cause spontaneous dissociative d-glucose adsorption, leading to the breaking of the one O1-C ring bond, the transfer of the hydrogen atom H2 from O2 in the sugar molecule to O_S1_ in the TiO_2_ surface (highlighted in yellow in Figure 6A), and the consequent formation of a carbonyl group. Furthermore, H2 establishes a hydrogen bond with O_S3_, whose calculated value is 1.843Å. The ether oxygen O1 of the glucose molecule binds to Ti (5f)_c_ atom and the calculated distance is 1.903Å. O6Ti (5f)_e_ interaction is calculated to be 2.269Å. The H6-O_S2_ hydrogen bond is 1.828Å, and H3-O(H)_s_ is 1.788Å. However, in the cluster containing a subsurface vacancy, non-dissociative adsorption of d-glucose occurs as pointed out in Figure 6B. Interestingly, the sugar molecule is moved during the optimization to one side of the cluster, and the optimized structure is stabilized by the formation of four hydrogen bonds: H4---O_S2_, 1.643Å; H_B2_---O4, 1.843Å; H2---O(H)_S,_ 1.588Å; H3---O_B1_, 2.185Å. Therefore, the presence of surface oxygen vacancies creates uncoordinated Ti species that are conveniently coordinated by the oxygen atoms of the adsorbed molecule. As a consequence, the overall distortion of the cluster is not significant. On the other hand, the formation of a subsurface oxygen vacancy leads to a higher cluster distortion since the bond distance between the two Ti (5f)_c_ and the corresponding O_S1_-Ti-O_S1’_ angle decreases (see Figure 5A for labels reference).

The relative adsorption energies can be calculated as in Eq. 8:

$$E{\left(V_{O}\right)}_{ads}=E{\left(V_{O}\right)}_{cluster+D-glucose}-\;E{\left(V_{O}\right)}_{cluster}-E_{D-glucose},$$ (9)

where $E{\left(V_{O}\right)}_{cluster+D-glucose}$ is the energy of the d-glucose adsorbed on the O-defective cluster structure, $E{\left(V_{O}\right)}_{cluster}$ is the energy of the O-defective cluster, and $E_{D-glucose}$ is the energy of the d-glucose molecule. From the results reported in Table 1 and 6, we can see that the adsorption of d-glucose on the cluster containing an oxygen subsurface vacancy is 0.24 and 0.40 eV more favored than that occurring on the stoichiometric anatase and on anatase with a surface oxygen vacancy, respectively. Selloni and co-workers (2010) have performed DFT calculations to elucidate the role of subsurface defects in the adsorption and dissociation of water on the anatase (101) surfaces. In analogy with our cluster results, the authors find that the presence of subsurface oxygen defects leads to stronger binding energies. Moreover, molecular adsorption is found to be favored than dissociative adsorption on most of the investigated sites.

**TABLE 6:** Adsorption energies of d-glucose on cluster-containing subsurface, $\text{E}{\left({\text{V}}_{\text{O}}\right)}_{\text{ads}}^{\text{sub}}$, and surface, $\text{E}{\left({\text{V}}_{\text{O}}\right)}_{\text{ads}}^{\text{surf}}$, oxygen vacancy calculated at B3LYP and B3LYP-D3BJ level of theory. Values are given in eV.

	B3LYP	B3LYP-D3BJ
$E{\left(V_{O}\right)}_{\mathrm{ads}}^{\mathrm{sub}}$	−2.73	−3.37
$E{\left(V_{O}\right)}_{\mathrm{ads}}^{\mathrm{surf}}$	−2.33	−3.23

In order to further check the reliability of those results, we have performed the calculations taking into account the dispersion correction. Our results show that the inclusion of dispersion does not affect the final geometry of the optimized structures in terms of moleculecluster interactions. Furthermore, the trend of adsorption energies is found to be very similar to the previous case, with the O1-center ads confirmed as the most favored coordination mode. The calculated value corresponds to -3.01 eV (−2.49 eV without dispersion, see Table 1). When calculations are done including dispersion corrections, the formation of a subsurface oxygen vacancy is also found to be favored than the formation of a surface oxygen vacancy, as reported in Table 5. Furthermore, adsorption of d-glucose on the cluster with a subsurface oxygen vacancy is also preferred (see Table 6).

Our results have therefore stated that the presence of defects, such as surface and subsurface oxygen vacancies, leads to stronger adsorption energies that favor the adsorption of d-glucose on the TiO_2_ surface, thus confirming the important role of defects in the surface chemistry of TiO_2_.

### Electronic Structure Analysis

**FIGURE 7:**
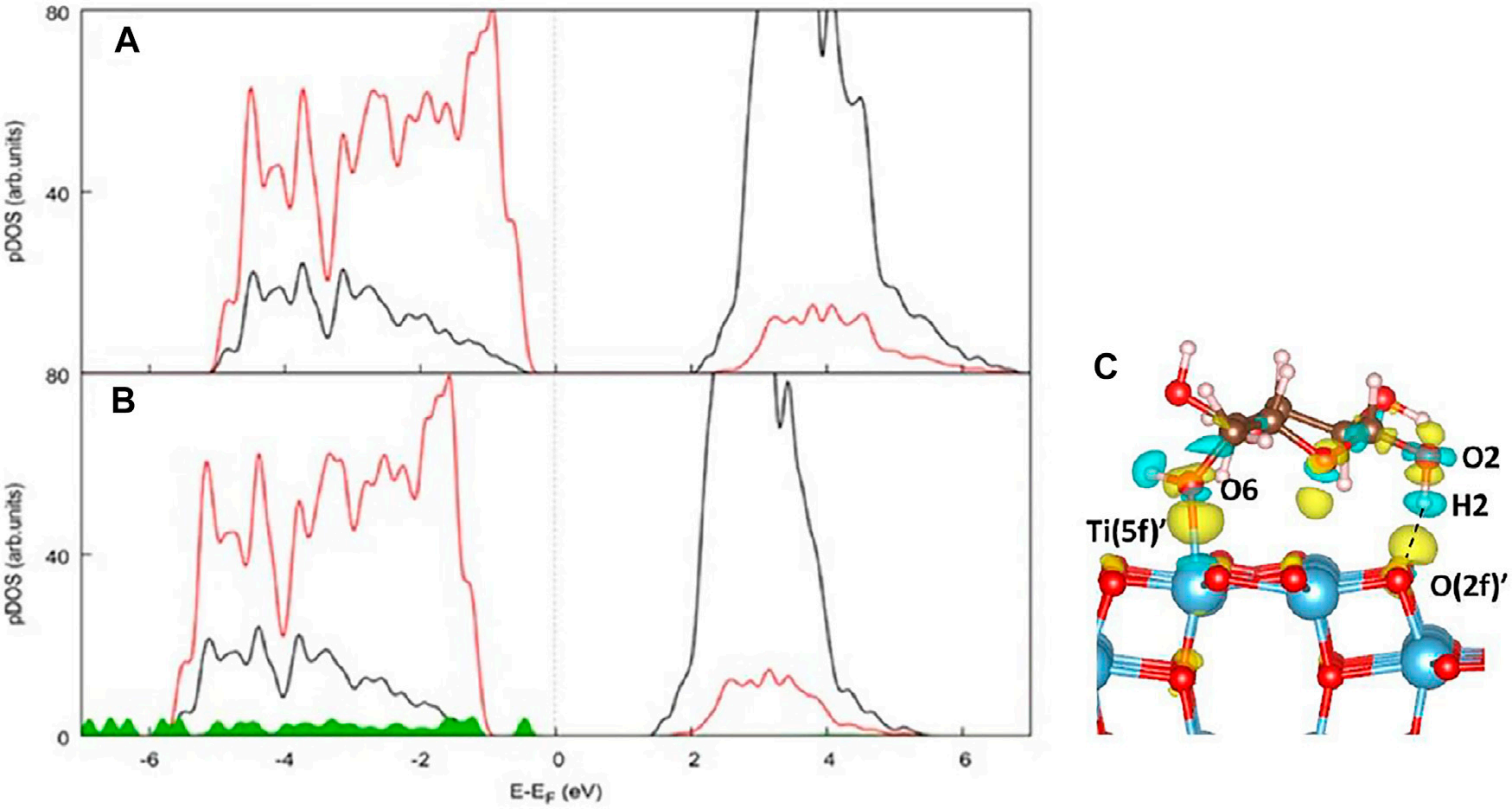
Atom- and angular momentum-projected density of states (pDOS) of **(A)** the TiO_2_ (100) surface and **(B)** the d-glucose-TiO_2_ (100) system in the most stable configuration Ads_1 computed at the PBE + U-D3BJ level of theory. Color code: Ti d states black, O p states red, Glucose states green. **(C)** Charge density difference plot referred to Ads_1. Color code: yellow and cyan areas indicate an electron accumulation and depletion, respectively. The isosurface level is set to 0.003 eV/Å^3^.

We report the projected density of states (pDOS) of the (100) TiO_2_ surface before (Figure 7A) and after glucose adsorption as in Ads_1 (Figure 7B). In the pristine (100) surface, the valence band mainly consists of O 2p states, while the Ti d states mainly contribute to the conduction band. However, the nature of the valence band is modified due to the contribution of glucose states. As a consequence, the calculated values of the band gaps are also very different and correspond to 2.09eV for the bare TiO_2_ (100) and 1.462eV for the whole system TiO_2_-d-glucose. The calculated band gap for the bare TiO_2_ surface is much smaller than the experimental gap (∼3.2 eV). However, it is known that DFT calculations tend to underestimate the band gaps of metal oxides. In Figure 7C, the charge density difference plot related to Ads_1 is shown, which underlines the presence of charge density in the region between O2H2 and O (2f)' associated with the formation of a hydrogen bond as discussed above. Moreover, charge density is also localized between O6 and Ti (5f), and all the atoms directly bound to Ti, confirming the formation of a strong O (glucose)-Ti (anatase) bond.

Using the cluster model approach, band gaps can be approximated to HOMO-LUMO gaps. Table 7 shows the HOMOLUMO gaps computed at B3LYP-D3BJ level of theory for the selected cluster model with and without d-glucose.

**TABLE 7:** HOMOLUMO gap energies (HL gap) calculated at B3LYP-D3BJ for the cluster models of both bare (100) TiO_2_ surface and adsorbed d-glucose in O1-center mode. All the energies are given in eV.

	Bare cluster	Cluster + D-glucose
**HL gap**	4.51	4.45

The calculated HL gap for the stoichiometric TiO_2_ cluster model is 4.51eV. The adsorption of d-glucose slightly changes the HL gap lowering it by 0.06eV. Lunell and co-workers (Balducci, 2010) have calculated a HL gaps of 4.98 and 4.94eV using a (TiO_2_)_16_ cluster at B3LYP/VDZ and B3LYP/V(T)DZ methods. HL gaps of 4.62 and 4.14 eV have been calculated by the authors using a much smaller cluster (TiO_2_)_5_ at B3LYP/VDZ and B3LYP/6–31G (d,p) level. All those results underline how the size of the cluster models along with the used computational method has large influence on the HL gap values. Moreover, the CA at the DFT level tends to give HL gaps that are much higher than the experimental band gap.

## Conclusion

This work reports the computational study of a medium-to-large and complex molecule such as d-glucose adsorbed on the anatase TiO_2_ (100) surface, offering a direct comparison between two different and complementary computational methods. Four different adsorption modes have been investigated at the B3LYP level of theory, considering also the inclusion of the dispersion correction D3BJ. The most favored adsorption mode is the O1-center-ads where the d-glucose adsorbs via the formation of H-bond between its hydroxyl groups and the oxygen atoms of the anatase surface, confirming the importance of hydrogen bond formation in the adsorption modes.

We also considered the formation of surface and subsurface oxygen vacancies in our cluster model approach. Our results with B3LYP and B3LYP-D3BJ show surface and subsurface oxygen formation energies that are higher than those calculated by Selloni and co-workers (2010) using PBC calculations. Nevertheless, both the computational methods confirm that the formation energy of O vacancies is lower in the subsurface than at the surface. Regarding the interaction of d-glucose with these defective TiO_2_ (100) surfaces, our results show that the presence of defects, both surface and subsurface oxygen vacancies, provides stronger adsorption energies that favor the d-glucose on binding to TiO_2_, thus confirming the important role of defects in the titania surface chemistry. Since realistic TiO_2_ films that contain defects or oxygen vacancies, for example, due to non-ideal sputter deposition processes, our finding supports the role of the anatase (100) as efficient sensor surface, which can accumulate analyte molecule.

In analogy to our first cluster model, Ads_1 is found to be the most favored adsorption mode. However, PBC calculations show the formation of the Ti-O bond, involving an oxygen atom of a hydroxyl group of d-glucose with a five-fold coordinated Ti atom, Ti (5f), which is not observed in cluster investigations. The calculated PBC adsorption energies are significantly less favorable than those computed with cluster models. However, CP corrections have been calculated, and they show that these discrepancies are mostly due to BSSE arising from the use of localized basis sets in cluster model calculations.

Our results have pointed out that the selected medium-size cluster model is able to properly describe the adsorption of the big and complex d-glucose molecule and to reproduce the influence of defects in the adsorption. Those outcomes contribute to enlarge the understanding of the interaction and binding mode between the analyte and the surface which is a key aspect for the design and development of innovative biosensors. Moreover, the proposed cluster models can be used for further investigation of reaction mechanisms as, for example, in TiO_2_-based catalytic biomass conversion, allowing the identification of key structures, such as transition states and intermediates, and for the study of charged systems at a lower computational cost than PBC calculations. Furthermore, we have focused our attention on anatase (100) termination, for which much less information is available in the literature. However, enriching the knowledge about this surface can be fundamental for the application of TiO_2_ in several fields, including the photocatalytic conversion of biomass. As future work, we will investigate the 1) effect of d-glucose coverage on anatase (100) surface, considering the presence of a further sugar molecule on our selected clusters and the potential interaction of d-glucose molecules in PBC calculations; 2) solvent effect by a direct comparison of implicit and explicit solvent models.

## Data Availability

The original contributions presented in the study are included in the article/Supplementary Material; further inquiries can be directed to the corresponding author.

## References

[B1] Abbasi A. Sardroodi J. J. (2018). Exploration of Sensing of Nitrogen Dioxide and Ozone Molecules Using Novel TiO_2_/Stanene Heterostructures Employing DFT Calculations. Appl. Surf. Sci. 442, 368–381. 10.1016/j.apsusc.2018.02.183

[B2] Abrahams J. Davidson R. S. Morrison C. L. (1983). Optimization of the Photocatalytic Properties of Titanium Dioxide. J. Phys. Chem. 87, 353–361.

[B3] Adamo C. Barone V. (1999). Toward Reliable Density Functional Methods without Adjustable Parameters: The PBE0 Model. J. Chem. Phys. 110, 6158–6170. 10.1063/1.478522

[B4] Andrae D. Häußermann U. Dolg M. Stoll H. Preuß H. (1990). Energy-adjustedab Initio Pseudopotentials for the Second and Third Row Transition Elements. Theoret. Chim. Acta 77, 123–141. 10.1007/bf01114537

[B5] Aschauer U. He Y. Cheng H. Li S.-C. Diebold U. Selloni A. (2010). Influence of Subsurface Defects on the Surface Reactivity of TiO_2_: Water on Anatase (101). J. Phys. Chem. C 114, 1278–1284. 10.1021/jp910492b

[B6] Bai Y. Mora-Seró I. De Angelis F. Bisquert J. Wang P. (2014). Titanium Dioxide Nanomaterials for Photovoltaic Applications. Chem. Rev. 114, 10095–10130. 10.1021/cr400606n 24661129

[B7] Balducci G. (2010). The Adsorption of Glucose at the Surface of Anatase: A Computational Study. Chem. Phys. Lett. 494, 54–59. 10.1016/j.cplett.2010.05.068

[B8] Bandura A. V. Sykes D. G. Shapovalov V. Troung T. N. Kubicki J. D. Evarestov R. A. (2004). Adsorption of Water on the TiO_2_(Rutile) (110) Surface: A Comparison of Periodic and Embedded Cluster Calculations. J. Phys. Chem. B 108 (23), 7844–7853. 10.1021/jp037141i

[B9] Bao S.-J. Li C. M. Zang J.-F. Cui X.-Q. Qiao Y. Guo J. (2008). New Nanostructured TiO_2_ for Direct Electrochemistry and Glucose Sensor Applications. Adv. Funct. Mater. 18, 591–599. 10.1002/adfm.200700728

[B11] Bella F. Muñoz-García A. B. Colò F. Meligrana G. Lamberti A. Destro M. (2018). Combined Structural, Chemometric, and Electrochemical Investigation of Vertically Aligned TiO_2_ Nanotubes for Na-Ion Batteries. ACS Omega 3, 8440–8450. 10.1021/acsomega.8b01117 31458972 PMC6644502

[B10] Bella F. Muñoz-García A. B. Meligrana G. Lamberti A. Destro M. Pavone M. (2017). Unveiling the Controversial Mechanism of Reversible Na Storage in TiO2 Nanotube Arrays: Amorphous versus Anatase TiO_2_ . Nano Res. 10, 2891–2903. 10.1007/s12274-017-1656-6

[B12] Burdett J. K. Hughbanks T. Miller G. J. Richardson J. W. Smith J. V. (1987). Structural-Electronic Relationships in Inorganic Solids: Powder Neutron Diffraction Studies of the Rutile and Anatase Polymorphs of Titanium Dioxide at 15 and 295 K. J. Am. Chem. Soc. 109 (12), 3639–3646. 10.1021/ja00246a021

[B13] Butera V. Caspary Toroker M. (2016). Electronic Properties of Pure and Fe-Doped β-Ni(OH)2: New Insights Using Density Functional Theory with a Cluster Approach. J. Phys. Chem. C 120, 12344–12350. 10.1021/acs.jpcc.6b01501

[B15] Butera V. Fukaya N. Choi J.-C. Sato K. Choe Y.-K. (2018). Alkoxysilane Production from Silica and Dimethylcarbonate Catalyzed by Alkali Bases: A Quantum Chemical Investigation of the Reaction Mechanism. Inorg. Chim. Acta 482, 70–76. 10.1016/j.ica.2018.05.036

[B16] Butera V. Tanabe Y. Miyazawa T. Fujitani T. Kayanuma M. Choe Y.-K. (2021). Mechanistic Investigation on Ethanol-To-Butadiene Conversion Reaction over Metal Oxide Clusters. Int. J. Quan. Chem. 121, e26494. 10.1002/qua.26494

[B14] Butera V. Toroker M. C. (2017). Practical Cluster Models for a Layered β-NiOOH Material. Materials 10, 480. 10.3390/ma10050480 PMC5459011 28772839

[B17] Chang J.-G. Ju S.-P. Chang C.-S. (2008). A Computational Study on Adsorption Configurations and Dissociative Reactions of the HN3 Molecule on the TiO_2_ Anatase (101) Surface. J. Phys. Chem. C 112, 18017–18027. 10.1021/jp8050559

[B18] Cheng H. Selloni A. (2009). Surface and Subsurface Oxygen Vacancies in Anatase TiO_2_ and Differences with Rutile. PHYSICAL REVIEW B 79, 092101–092104. 10.1103/physrevb.79.092101

[B19] D’Arienzo M. Gamba L. Morazzoni F. Cosentino U. Greco C. Lasagni M. (2017). Experimental and Theoretical Investigation on the Catalytic Generation of Environmentally Persistent Free Radicals from Benzene. J. Phys. Chem. C 121, 9381–9393. 10.1021/acs.jpcc.7b01449

[B20] De Angelis F. Di Valentin C. Fantacci S. Vittadini A. Selloni A. (2014). Theoretical Studies on Anatase and Less Common TiO_2_ Phases: Bulk, Surfaces, and Nanomaterials. Chem. Rev. 114, 9708–9753. 10.1021/cr500055q 24926899

[B21] Doong R.-a. Shih H.-m. (2010). Array-based Titanium Dioxide Biosensors for Ratiometric Determination of Glucose, Glutamate and Urea. Biosens. Bioelectron. 25, 1439–1446. 10.1016/j.bios.2009.10.044 19954963

[B22] Dudarev S. L. Botton G. A. Savrasov S. Y. Humphreys C. J. Sutton A. P. (1998). Electron-energy-loss Spectra and the Structural Stability of Nickel Oxide: An LSDA+U Study. Phys. Rev. B 57, 1505–1509. 10.1103/physrevb.57.1505

[B23] Dudarev S. L. Botton G. A. Savrasov S. Y. Humphreys C. J. Sutton A. P. (1998). Electron-Energy-Loss Spectra and the Structural Stability of Nickel Oxide: An LSDA+U Study. Phys. Rev. B 57, 1505–1509. 10.1103/physrevb.57.1505

[B24] Finazzi E. Di Valentin C. Pacchioni G. Selloni A. (2008). Excess Electron States in Reduced Bulk Anatase TiO2: Comparison of Standard GGA, GGA+U, and Hybrid DFT Calculations. J. Chem. Phys. 129, 154113–154119. 10.1063/1.2996362 19045182

[B25] Foster J. P. Weinhold F. (1980). Natural Hybrid Orbitals. J. Am. Chem. Soc. 102, 7211–7218. 10.1021/ja00544a007

[B26] Grimme S. Antony J. Ehrlich S. Krieg H. (2010). A Consistent and Accurate Ab Initio Parametrization of Density Functional Dispersion Correction (DFT-D) for the 94 Elements H-Pu. J. Chem. Phys. 132, 154104–154119. 10.1063/1.3382344 20423165

[B27] Grimme S. Ehrlich S. Goerigk L. (2011). Effect of the Damping Function in Dispersion Corrected Density Functional Theory. J. Comput. Chem. 32, 1456–1465. 10.1002/jcc.21759 21370243

[B28] Grimme S. (2006). Semiempirical GGA-type Density Functional Constructed with a Long-Range Dispersion Correction. J. Comput. Chem. 27, 1787–1799. 10.1002/jcc.20495 16955487

[B29] Guo Q. Zhou C. Ma Z. Yang X. (2019). Fundamentals of TiO_2_ Photocatalysis: Concepts, Mechanisms, and Challenges. Adv. Mater. 31, 1901997–1902022. 10.1002/adma.201901997 31423680

[B30] Islam M. M. Calatayud M. Pacchioni G. (2011). Hydrogen Adsorption and Diffusion on the Anatase TiO_2_(101) Surface: A First-Principles Investigation. J. Phys. Chem. C 115 (14), 6809–6814. 10.1021/jp200408v

[B31] Kapilashrami M. Zhang Y. Liu Y.-S. Hagfeldt A. Guo J. Probing the Optical Property and Electronic Structure of TiO_2_ Nanomaterials for Renewable Energy Applications Chem. Rev. 2014, 114, 9662–9707. 10.1021/cr5000893 25137023

[B32] Keith J. A. Muñoz-García A. B. Lessio M. Carter E. A. (2015). Cluster Models for Studying CO_2_ Reduction on Semiconductor Photoelectrodes. Top. Catal. 58, 46–56. 10.1007/s11244-014-0341-1

[B33] Kresse G. Furthmüller J. (1996). Efficient Iterative Schemes Forab Initiototal-Energy Calculations Using a Plane-Wave Basis Set. Phys. Rev. B 54, 11169–11186. 10.1103/physrevb.54.11169 9984901

[B34] Kuo C.-H. Poyraz A. S. Jin L. Meng Y. Pahalagedara L. Chen S.-Y. (2014). Heterogeneous Acidic TiO_2_ Nanoparticles for Efficient Conversion of Biomass Derived Carbohydrates. Green. Chem. 16, 785–791. 10.1039/c3gc40909k

[B35] Lazzeri M. Vittadini A. Selloni A. (2001). Structure and Energetics of stoichiometricTiO_2_ anatase Surfaces. Phys. Rev. B 63, 155409–9. 10.1103/physrevb.63.155409

[B36] Lettieri S. Gargiulo V. Alfè M. Amati M. Zeller P. Maraloiu V.-A. (2020). Simple Ethanol Refluxing Method for Production of Blue-Colored Titanium Dioxide with Oxygen Vacancies and Visible Light-Driven Photocatalytic Properties. J. Phys. Chem. C 124, 3564–3576. 10.1021/acs.jpcc.9b08993

[B37] Linsebigler A. L. Lu G. Yates J. T. Jr. (1995). Photocatalysis on TiO_2_ Surfaces: Principles, Mechanisms, and Selected Results. Chem. Rev. 95, 735–758. 10.1021/cr00035a013

[B38] Lundqvist M. J. Nilsing M. Persson P. Lunell S. (2006). DFT Study of Bare and Dye-Sensitized TiO_2_ Clusters and Nanocrystals. Int. J. Quan. Chem. 106, 3214–3234. 10.1002/qua.21088 17034238

[B39] Maity P. Mohammed O. F. Katsiev K. Idriss H. (2018). Study of the Bulk Charge Carrier Dynamics in Anatase and Rutile TiO_2_ Single Crystals by Femtosecond Time-Resolved Spectroscopy. J. Phys. Chem. C 122, 8925–8932. 10.1021/acs.jpcc.8b00256

[B40] Manera M. G. Spadavecchia J. Buso D. de Julián Fernández C. Mattei G. Martucci A. (2008). Optical Gas Sensing of TiO_2_ and TiO_2_/Au Nanocomposite Thin Films. Sensors Actuators B: Chem. 132, 107–115. 10.1016/j.snb.2008.01.014

[B41] Martinez-Casado R. Mallia G. Harrison N. M. Pérez R. (2018). First-Principles Study of the Water Adsorption on Anatase(101) as a Function of the Coverage. J. Phys. Chem. C 122 (36), 20736–20744. 10.1021/acs.jpcc.8b05081

[B42] Massaro A. Muñoz-García A. B. Maddalena P. Bella F. Meligrana G. Gerbaldi C. (2020). First-principles Study of Na Insertion at TiO_2_ Anatase Surfaces: New Hints for Na-Ion Battery Design. Nanoscale Adv. 2, 2745–2751. 10.1039/d0na00230e PMC9417436 36132399

[B43] Mino L. Spoto G. Ferrari A. M. (2014). CO_2_ Capture by TiO_2_ Anatase Surfaces: A Combined DFT and FTIR Study. J. Phys. Chem. C 118, 25016–25026. 10.1021/jp507443k

[B45] Neese F. (2017). Software Update: the ORCA Program System, Version 4.0, Wiley Interdiscip. Rev. Comput. Mol. Sci. 8, e1327. 10.1002/wcms.1327

[B44] Neese F. (2012). The ORCA Program System. Wires Comput. Mol. Sci. 2, 73–78. 10.1002/wcms.81

[B46] Onal I. Soyer S. Senkan S. (2006). Adsorption of Water and Ammonia on TiO2-Anatase Cluster Models. Surf. Sci. 600, 2457–2469. 10.1016/j.susc.2006.04.004

[B47] Paunesku T. Rajh T. Wiederrecht G. Maser J. Vogt S. Stojićević N. (2003). Biology of TiO_2_-Oligonucleotide Nanocomposites. Nat. Mater 2, 343–346. 10.1038/nmat875 12692534

[B48] Perdew J. P. Burke K. Ernzerhof M. (1996). Generalized Gradient Approximation Made Simple. Phys. Rev. Lett. 77, 3865–3868. 10.1103/physrevlett.77.3865 10062328

[B49] Perdew J. P. Burke K. Ernzerhof M. (1997). Generalized Gradient Approximation Made Simple [Phys. Rev. Lett. 77, 3865 (1996)]. Phys. Rev. Lett. 78, 1396. 10.1103/physrevlett.78.1396 10062328

[B50] Piccolo L. Afanasiev P. Morfin F. Len T. Dessal C. Rousset J. L. (2020). Operando X-ray Absorption Spectroscopy Investigation of Photocatalytic Hydrogen Evolution over Ultradispersed Pt/TiO_2_ Catalysts. ACS Catal.. 10, 12696–12705. 10.1021/acscatal.0c03464

[B51] Qiu J. Zhang S. Zhao H. (2011). Recent Applications of TiO_2_ Nanomaterials in Chemical Sensing in Aqueous media. Sensors actuators B: Chem. 160, 875–890. 10.1016/j.snb.2011.08.077

[B52] Ranade M. R. Navrotsky A. Zhang H. Z. Banfield J. F. Elder S. H. Zaban A. (2002). Energetics of Nanocrystalline TiO_2_ . Proc. Natl. Acad. Sci. 99, 6476–6481. 10.1073/pnas.251534898 11880610 PMC128553

[B53] Srivastava S. Ali M. A. Solanki P. R. Chavhan P. M. Pandey M. K. Mulchandani A. (2013). Mediator-free Microfluidics Biosensor Based on Titania-Zirconia Nanocomposite for Urea Detection. RSC Adv. 3, 228–235. 10.1039/c2ra21461j

[B54] Triggiani L. Muñoz-García A. B. Agostiano A. Pavone M. (2015). First-principles Study of Trimethylamine Adsorption on Anatase TiO_2_ Nanorod Surfaces. Theor. Chem. Acc. 134, 119. 10.1007/s00214-015-1721-8

[B55] van Putten R.-J. van der Waal J. C. de Jong E. Rasrendra C. B. Heeres H. J. de Vries J. G. (2013). Hydroxymethylfurfural, A Versatile Platform Chemical Made from Renewable Resources. Chem. Rev. 113, 1499–1597. 10.1021/cr300182k 23394139

[B56] Wang C. Yin L. Zhang L. Qi Y. Lun N. Liu N. (2010). Large Scale Synthesis and Gas-Sensing Properties of Anatase TiO2Three-Dimensional Hierarchical Nanostructures. Langmuir 26 (15), 12841–12848. 10.1021/la100910u 20597492

[B57] Wang Y. Zu M. Zhou X. Lin H. Peng F. Zhang S. (2020). Designing Efficient TiO_2_-Based Photoelectrocatalysis Systems for Chemical Engineering and Sensing. Chem. Eng. J. 381, 1226052. 10.1016/j.cej.2019.122605

[B58] Wu S. Weng Z. Liu X. Yeung K. W. K. Chu P. K. (2014). Functionalized TiO_2_ Based Nanomaterials for Biomedical Applications. Adv. Funct. Mater. 24, 5464–5481. 10.1002/adfm.201400706

[B59] Yu J. Low J. Xiao W. Zhou P. Jaroniec M. (2014). Enhanced Photocatalytic CO2-Reduction Activity of Anatase TiO_2_ by Coexposed {001} and {101} Facets. J. Am. Chem. Soc. 136, 8839–8842. 10.1021/ja5044787 24918628

[B60] Zhang H. Banfield J. F. (1998). Thermodynamic Analysis of Phase Stability of Nanocrystalline Titania. J. Mater. Chem. 8, 2073–2076. 10.1039/a802619j

