# Supplementary material for: d-Glucose Adsorption on the TiO2 Anatase (100) Surface: A Direct Comparison Between Cluster-Based and Periodic Approaches
Source: Front Chem. 2021 Aug 31;9:716329. doi: 10.3389/fchem.2021.716329 (PMC8438178; doi:10.3389/fchem.2021.716329)
Supplement: Supplementary file 1 [file DataSheet1.PDF]

Supplementary Material

D-glucose adsorption on TiO_2 anatase (100) surface: a direct comparison between cluster-based and periodic approaches

Valeria Butera,[1]* Arianna Massaro,[2] Ana B. Muñoz-García,[3] Michele Pavone,[4] and Hermann Detz[1,4]

[1] CEITEC - Central European Institute of Technology Central European Institute of Technology, Brno University of Technology, Purkyňova 123, Brno 612 00, Czech Republic. E-mail: butera@vutbr.cz
[2]Department of Chemical Sciences, Università di Napoli Federico II, Comp. Univ. Monte Sant'Angelo, via Cintia 21, 80126 Naples, Italy.
[3]Department of Physics "Ettore Pancini", Università di Napoli Federico II, Comp. Univ. Monte Sant'Angelo, via Cintia 21, 80126 Naples, Italy.
[4] Center for Micro- and Nanostructures & Institute of Solid State Electronics, TU Wien, 1040, Vienna, Austria.

S1. Cluster Model Approach using PBE0

S2. Cartesian coordinates of all the optimized cluster structures

S1. Cluster Model Approach using PBE0

To further test the reliability of B3LYP functional, we have performed calculations on cluster models using PBE0[i] functional in combination with dispersion corrections and def2-TZVP basis set has been also employed. In this case, the RIJCOSX approximation was used, which includes RI-J is used for the Coulomb integrals and numerical chain-of-sphere integration for the HF Exchange integrals (COSX), to speed up the calculation's runtime, as reported in ORCA manual.
Moreover, a different cluster model, named **cluster b**, has been selected and investigated. In the next subsection S.1.1. we will present the results involving the cluster discussed in the main text, here referred as **cluster a**, while in subsection S.1.2. we will give a description of the new selected cluster and we will discuss the related results.

S.1.1. Cluster a.

In order to have a direct comparison with the periodic boundary condition calculations that we will discuss in the next section, our investigation has been performed using PBE0 functional. Optimized cluster structure performed using PBE0 shows the same distortion seen in the calculation done using B3LYP functional: the O_{S1}-$O_{S1'}$ distance is 2.822 Å; as a consequence of the shorter oxygen distance, the O_{S1}-Ti- $O_{S1'}$ decreases significantly to 107.1. Based on our previous results, we have investigated only the **O1-center ads** mode. Our results show that, in comparison with B3LYP functional, in this case an additional hydrogen bond, H4-O_{S2}, is formed whose computed value is 1.976 Å. The computed adsorption energy is -1.63 eV.
Results obtained with PBE0 confirm that the formation of a subsurface oxygen vacancy is favored than that of a surface oxygen vacancy. The calculated values are 4.76 eV and 5.43 eV, respectively (see table 3). Those values are lower than those calculated using B3LYP/D3 functional and closer to the ones, discussed above, calculated by Selloni and co-workers[ii] using (101) and (001) slab models and PBE functional.

S.1.2. Cluster b. A different cluster model has been selected in order to take into account the adsorption of D-glucose on the gap formed on the (100) surface between two rows of the building blocks. The optimized structure, shown in figure S1, contains 76 atoms and was obtained keeping fixed the position of all the outmost oxygen atoms. Calculations were done using PBE0 functional, def2-TZVP basis set and the RIJCOSX approximation.

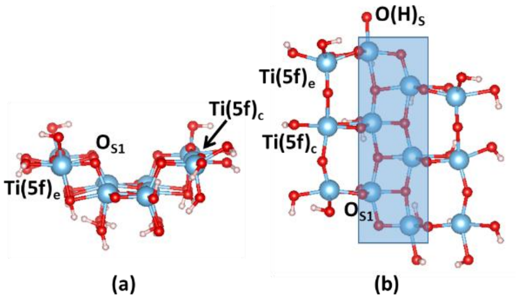

Figure S1. Optimized cluster structure that takes into account the step on 100 surface. O: red; H: white; Ti: blue.

Since our previous results have shown that more favorable adsorption energies are computed when the D-glucose molecule keeps an orientation that is parallel to the TiO2 surface, only this situation has

been investigated as shown in figure S2.

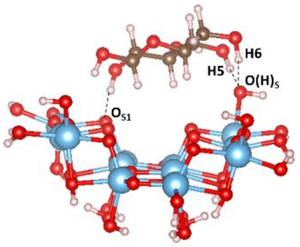

Figure S2. Optimized structure of D-glucose adsorbed on the second selected cluster model.

The optimized structure underlines the formation of three hydrogen bonds: the first one is established between H3 and the oxygen atom Os1 of TiO2, whose calculated distance is 1.70271 Å; O(H)s establishes two hydrogen bonds with H5 and H6 whose distances are 1.56276 and 1.67867 Å, respectively. The computed adsorption energy is -4.97 eV, which is 3.35 eV more favorable than the adsorption energy of D-glucose on the cluster model a at the same level of theory.
The “exaggerated” adsorption energy value indicates the weakness of the cluster model b in modeling D-glucose adsorption on TiO2 surface. Even though cluster of similar and smaller size have been reported in literature, they were related to the adsorption studies of smaller molecules, i.e. water and ammonia. However, our results have shown that the “one layer” cluster model fails to properly describe the adsorption energy of bigger molecule, such as D-glucose. We do believe that the inclusion of a second layer or the extension of the “one layer” surface is necessary requirement to obtain more reliable results. However, because of the computational cost of considering a bigger cluster model, this investigation was not performed at this stage of our investigation.

S2. Cartesian coordinates of all the optimized cluster structures

Bare cluster as in figure 1
68
Coordinates from ORCA-job E=-4201.157845895785 hartree

Ti	-9.277842	13.205658	-4.449417
Ti	-11.317513	13.100454	-6.716709
O	-9.208867	13.176631	-2.606289
O	-10.972950	13.732674	-4.883114
O	-11.330631	12.808398	-8.686102
O	-9.369172	13.180512	-6.708002
O	-9.252433	11.390779	-10.381215
Ti	-11.056022	11.395027	-9.763051
O	-11.856287	11.355435	-6.363116
Ti	-9.291630	9.438485	-4.480148
Ti	-11.321907	9.619999	-6.731835
Ti	-9.014598	11.289473	-2.117324
O	-9.222153	9.447775	-2.625090
O	-10.997535	8.940018	-4.924726
O	-9.162394	11.317172	-4.283763
O	-11.300311	9.970763	-8.716689
O	-9.382081	9.524896	-6.722988
Ti	-5.652412	13.195084	-4.657573
Ti	-7.539095	13.199177	-7.201127

O	-5.627633	13.230947	-2.862417
O	-7.495580	13.748030	-5.073478
O	-7.632769	13.288748	-9.000240
O	-5.678103	13.708975	-6.821702
O	-6.156902	11.357008	-10.639722
Ti	-7.645412	11.367009	-9.561236
O	-7.300893	11.346723	-7.424897
Ti	-5.664837	9.425314	-4.688074
Ti	-7.550339	9.492673	-7.231003
Ti	-5.657593	11.293348	-2.366544
O	-5.650801	9.369355	-2.891706
O	-7.519337	8.892212	-5.108950
O	-5.832346	11.310993	-4.486356
O	-7.663070	9.433497	-9.028361
O	-5.703500	8.955655	-6.859513
O	-7.276205	11.295428	-1.486828
Ti	-1.894870	13.046772	-5.079151
Ti	-3.897968	13.086854	-7.322643
O	-1.970104	12.685808	-3.101164
O	-3.822732	13.350858	-5.086238
O	-3.988845	12.804085	-9.227560
O	-2.190255	13.627348	-6.946385
Ti	-4.335696	11.346727	-10.255704
O	-3.961904	11.318726	-6.949835
Ti	-1.907631	9.528608	-5.090670
Ti	-3.906635	9.548982	-7.363397
Ti	-2.260566	11.263617	-2.056436
O	-1.956637	9.849974	-3.127869
O	-3.843146	9.248193	-5.108284
O	-1.380897	11.292749	-5.382153
O	-3.994047	9.855969	-9.246053
O	-2.214292	8.988148	-6.975629
O	-4.078057	11.275706	-1.476475
O	-12.671953	14.415801	-7.042626
H	-12.922403	14.435849	-7.987813
O	-12.298272	11.315317	-11.218480
H	-12.748712	10.508631	-11.520609
O	-12.675251	8.323502	-7.137453
H	-12.897622	8.327672	-8.089364
O	-0.538471	8.242988	-4.758014
H	-0.291537	8.209784	-3.812615
O	-3.288414	11.433838	-11.818712
H	-2.554611	12.022358	-12.053234
O	-1.054187	11.338062	-0.571664
H	-0.621385	12.147001	-0.250206
O	-0.514696	14.310224	-4.694114
H	-0.277968	14.316940	-3.745951

O	-10.248357	11.462036	-0.699642
H	-10.592634	12.364368	-0.555740

Bidentate adsorption of D-glucose as in figure 3
92
Coordinates from ORCA-job E= -4887.811029533869 hartree

O	13.112195	-12.615232	-5.786779
Ti	12.345854	-3.640624	-4.384878
Ti	13.492459	-6.297910	-6.358525
O	13.058281	-4.439149	-5.835020
O	13.561816	-8.036125	-7.309709
O	13.772727	-6.843615	-4.606305
Ti	11.670948	-11.411697	-5.688292
O	10.070455	-12.262906	-6.116236
O	11.524434	-9.260244	-5.392949
O	12.090740	-10.612440	-7.413556
Ti	11.905188	-8.784961	-7.193117
O	11.608877	-6.587042	-6.813185
Ti	9.088965	-4.359844	-5.066498
Ti	9.781660	-6.355963	-7.161933
O	9.568675	-4.578646	-6.996934
O	10.487605	-3.284658	-4.654306
O	10.169594	-8.313518	-7.993039
O	9.147678	-6.472459	-5.384467
Ti	8.386883	-11.575895	-6.368137
O	6.935308	-12.558521	-6.947194
O	8.303785	-9.492011	-6.102428
O	8.737284	-10.819882	-8.164431
Ti	8.333081	-9.024115	-8.013107
O	8.123693	-6.882125	-8.152331
Ti	5.774780	-4.544996	-5.704872
Ti	6.230719	-6.588220	-7.876981
O	5.989749	-4.763745	-7.596866
O	7.376921	-3.718889	-5.265957
O	6.448403	-8.807046	-8.039126
O	5.908339	-6.675894	-6.005899
Ti	5.115923	-12.222218	-7.275987
O	4.208552	-13.823213	-7.670318
O	3.702620	-8.997610	-6.760870
O	4.904415	-10.976132	-8.642140
Ti	4.483645	-9.055578	-8.409179
O	4.843831	-7.163420	-8.927798
O	4.327582	-3.339072	-5.600531
O	3.094050	-9.038355	-9.694012
Ti	12.873813	-6.837747	-2.984765
O	12.524551	-4.857226	-3.091446
O	12.742974	-8.757699	-2.631658
O	11.465975	-11.180127	-3.789513

Ti	11.258065	-9.355442	-3.528869
O	10.972314	-7.146657	-3.280484
Ti	9.118310	-6.915753	-3.549732
O	8.894745	-5.161795	-3.237535
O	9.328235	-9.080325	-3.325373
O	7.865905	-11.378612	-4.471569
Ti	7.707553	-9.584722	-4.320075
O	7.277331	-7.635028	-3.450019
Ti	5.532395	-7.162393	-4.209948
O	5.316482	-5.348958	-3.968875
O	5.845388	-9.389328	-4.608312
O	4.409952	-11.472941	-5.822556
Ti	4.027316	-9.696632	-5.063833
O	3.895016	-7.950745	-4.042284
O	2.548584	-10.271696	-4.004380
O	14.991538	-5.571331	-7.289580
H	15.085983	-4.607565	-7.155574
O	13.882915	-6.433557	-1.413309
H	13.927801	-5.473618	-1.235990
O	13.295565	-2.063922	-3.879720
H	13.589107	-1.832113	-2.983161
H	13.628415	-12.615102	-6.615497
H	3.821036	-3.360301	-4.765797
H	3.017935	-8.249017	-10.261124
H	2.240324	-9.567497	-3.400220
H	3.437109	-14.233963	-7.250718
O	7.859975	-12.662902	-12.891401
O	5.355815	-10.388526	-11.271563
O	7.872574	-9.342643	-10.288438
O	10.137499	-11.352183	-10.334404
O	10.051995	-13.195738	-12.338201
O	5.773652	-13.625452	-14.257993
C	6.627654	-10.824050	-11.795058
C	7.724416	-10.746914	-10.726050
C	6.534826	-12.238626	-12.393740
C	9.048212	-11.266553	-11.270571
C	8.830091	-12.688030	-11.811933
C	5.629318	-12.325521	-13.615863
H	6.870719	-10.123792	-12.603717
H	7.427814	-11.326442	-9.846450
H	6.203150	-12.950810	-11.623226
H	9.369811	-10.636158	-12.109733
H	8.448343	-13.345082	-11.012285
H	5.895431	-11.520744	-14.317888
H	4.585200	-12.209571	-13.323179
H	5.131995	-10.795581	-10.373157
H	6.980412	-8.923859	-10.289680

H	9.813747	-11.212709	-9.388604
H	10.748449	-12.836286	-11.737189
H	6.732551	-13.838046	-14.192871

Monodentate adsorption of D-glucose as in figure 3

92

Coordinates from ORCA-job E= -4887.775112421198 hartree

O	12.190235	-13.688804	-5.942702
Ti	12.550861	-4.293010	-5.128036
Ti	13.118981	-6.512370	-7.179962
O	12.832269	-4.653562	-6.966875
O	13.185020	-8.504109	-7.569514
O	13.181743	-6.475484	-5.271935
Ti	11.074812	-12.177432	-5.919291
O	9.283635	-12.660587	-6.259949
O	11.013140	-8.850731	-5.797046
O	11.552275	-11.039890	-7.227202
Ti	11.480751	-9.047463	-7.562505
O	11.298156	-6.899849	-7.786379
Ti	9.143703	-4.495717	-5.440128
Ti	9.421045	-6.581713	-7.541817
O	9.331697	-4.807635	-7.417228
O	10.758118	-3.685280	-5.213041
O	9.591692	-8.671838	-8.322256
O	9.166729	-6.573490	-5.636837
Ti	7.707058	-11.726967	-6.315862
O	6.167812	-12.657529	-6.576033
O	7.822766	-9.596087	-6.100951
O	7.844765	-11.012597	-8.192274
Ti	7.730368	-9.209265	-7.929016
O	7.670671	-7.057783	-8.231550
Ti	5.778315	-4.416635	-5.763815
Ti	5.780963	-6.589847	-7.855221
O	5.738419	-4.742585	-7.650443
O	7.474088	-3.705568	-5.539896
O	5.888143	-8.869723	-8.077017
O	5.741471	-6.601239	-5.963470
Ti	4.329761	-12.205434	-6.660287
O	3.221852	-13.735898	-6.764945
O	3.276459	-8.853163	-6.411231
O	4.065786	-11.002817	-7.996243
Ti	3.954250	-9.089265	-8.125536
O	4.217724	-7.093017	-8.588650
O	4.466225	-3.072232	-5.567468
O	2.684453	-9.034776	-9.542002
Ti	12.750342	-6.889779	-3.434947
O	12.547742	-4.968220	-3.369040
O	12.773874	-8.854080	-3.518955

O	11.164179	-11.233184	-4.359421
Ti	11.093306	-9.354076	-4.060474
O	10.863139	-7.267077	-3.294875
Ti	9.044799	-6.933046	-3.757177
O	8.934202	-5.149520	-3.574662
O	9.176816	-9.115549	-3.585026
O	7.459286	-11.383376	-4.363372
Ti	7.429856	-9.584552	-4.253517
O	7.227181	-7.523074	-3.444971
Ti	5.456116	-6.955406	-4.121550
O	5.417402	-5.114902	-3.968886
O	5.572966	-9.197803	-4.403338
O	3.877609	-11.277386	-5.197082
Ti	3.680448	-9.401188	-4.691610
O	3.786563	-7.572999	-3.780680
O	2.210001	-9.707110	-3.506701
O	14.677708	-6.164773	-8.220484
H	14.876408	-5.214378	-8.324935
O	13.977724	-6.776679	-1.970754
H	14.107485	-5.855585	-1.670446
O	13.751452	-2.861104	-4.792657
H	13.911992	-2.683300	-3.845755
H	12.857421	-13.989808	-6.577995
H	4.066573	-3.015636	-4.677846
H	2.595275	-8.126992	-9.895496
H	2.010102	-8.906105	-2.982427
H	2.488016	-13.972043	-6.174789
O	8.767784	-10.158436	-11.963041
O	8.864153	-13.910332	-11.875378
O	10.752335	-13.173723	-9.739223
O	11.673352	-10.489545	-9.675823
O	10.047750	-8.476743	-10.980014
O	7.051936	-10.600898	-14.056446
C	8.959981	-12.542653	-11.358640
C	10.349447	-12.252157	-10.787278
C	8.651788	-11.523735	-12.467653
C	10.384701	-10.809599	-10.279555
C	10.104433	-9.804854	-11.397718
C	7.234998	-11.610296	-13.023001
H	8.209871	-12.471729	-10.565023
H	11.107713	-12.389345	-11.577194
H	9.369916	-11.662761	-13.299239
H	9.602642	-10.727112	-9.522590
H	10.838167	-9.888032	-12.212830
H	6.514829	-11.471114	-12.206534
H	7.066852	-12.582832	-13.489578
H	9.662071	-14.096406	-12.413550

H	10.016969	-13.306303	-9.098942
H	11.809532	-11.136252	-8.919132
H	9.817521	-8.418764	-9.993656
H	7.318067	-9.750254	-13.642482

O1-edge adsorption of D-glucose as in figure 3

92

Coordinates from ORCA-job E= -4887.813261453945 hartree

O	12.319127	-13.485235	-5.341089
Ti	12.906856	-4.361947	-5.513036
Ti	13.425133	-7.448749	-7.102358
O	13.289379	-5.487480	-6.866250
O	13.166979	-9.320948	-7.668025
O	13.865268	-7.724828	-5.316609
Ti	11.092030	-12.061116	-5.289898
O	9.346668	-12.692195	-5.393833
O	11.233679	-9.890651	-5.371000
O	11.404914	-11.627570	-7.170872
Ti	11.450538	-9.785056	-7.250415
O	11.474665	-7.543109	-7.329996
Ti	9.528642	-4.701896	-5.671376
Ti	9.659613	-7.085247	-7.435271
O	9.705609	-5.294364	-7.564585
O	11.087666	-3.783221	-5.614636
O	9.652030	-9.240523	-7.865398
O	9.299515	-6.827215	-5.605218
Ti	7.713285	-11.873246	-5.485836
O	6.151953	-12.816638	-5.497307
O	7.912703	-9.737767	-5.555597
O	7.706160	-11.405156	-7.435365
Ti	7.794226	-9.614391	-7.432438
O	7.843447	-7.529714	-8.124978
Ti	6.159776	-4.503674	-5.905919
Ti	5.924773	-6.898811	-7.783254
O	5.988272	-5.024496	-7.762462
O	7.894053	-3.868582	-5.815069
O	5.939421	-9.115156	-7.670609
O	6.061797	-6.679784	-5.886048
Ti	4.344054	-12.277877	-5.619529
O	3.170858	-13.745140	-5.811559
O	3.394376	-8.930571	-5.744107
O	4.188699	-11.206228	-7.047353
Ti	4.023630	-9.295286	-7.415642
O	4.173853	-7.378829	-7.968693
O	4.934611	-3.075992	-5.841461
O	2.660185	-9.524167	-8.806738
Ti	13.219971	-7.324452	-3.629923
O	13.114237	-5.362316	-4.046579

O	12.906248	-9.131906	-2.915916
O	11.197620	-11.498816	-3.459958
Ti	11.250155	-9.639416	-3.494028
O	11.275883	-7.409666	-3.644239
Ti	9.440463	-6.950065	-3.726250
O	9.482057	-5.157444	-3.714111
O	9.426331	-9.047836	-3.060200
O	7.591981	-11.294077	-3.589980
Ti	7.625881	-9.495718	-3.702317
O	7.564559	-7.367235	-3.304943
Ti	5.803164	-6.813490	-3.997601
O	5.839666	-4.964596	-4.053135
O	5.779331	-9.110090	-3.810765
O	3.916610	-11.170412	-4.235642
Ti	3.879810	-9.268032	-3.962867
O	4.134075	-7.272323	-3.485889
O	2.534678	-9.221744	-2.617115
O	14.855742	-7.111138	-8.313667
H	15.077915	-6.162979	-8.392834
O	14.481528	-6.833248	-2.281255
H	14.678081	-5.876241	-2.280152
O	14.104616	-2.881597	-5.427992
H	14.542511	-2.526821	-4.637018
H	12.711042	-13.686791	-6.212357
H	4.534757	-2.872856	-4.975073
H	2.013049	-8.790698	-8.825828
H	2.442821	-8.318424	-2.252787
H	2.622208	-13.988275	-6.574162
O	5.863811	-6.744827	-10.251675
O	2.513503	-6.737409	-11.866034
O	3.037498	-9.504556	-11.524794
O	6.021074	-10.023316	-11.988756
O	7.712104	-8.196472	-10.609575
O	5.266417	-3.889936	-10.084060
C	3.641958	-7.216732	-11.059484
C	4.121997	-8.543365	-11.679028
C	4.708959	-6.127226	-10.970496
C	5.467292	-8.995359	-11.118703
C	6.448380	-7.814661	-11.092212
C	4.231457	-4.880007	-10.229756
H	3.314895	-7.416214	-10.034876
H	4.237258	-8.406984	-12.761510
H	5.078803	-5.840430	-11.964386
H	5.362777	-9.374186	-10.096771
H	6.569402	-7.409173	-12.106885
H	3.824234	-5.172985	-9.256040
H	3.424772	-4.446887	-10.831872

H	1.967277	-7.542636	-12.012257
H	2.928208	-9.705536	-10.544008
H	6.901713	-10.253946	-11.621855
H	7.855317	-7.890748	-9.641707
H	5.745460	-4.112850	-9.235848

O1-center adsorption of D-glucose as in figure 3
92
Coordinates from ORCA-job E= -4887.774375905489 hartree

O	12.632386	-13.258500	-5.547782
Ti	12.879545	-4.412558	-5.437378
Ti	13.328183	-6.921216	-7.218633
O	13.092927	-4.995411	-7.184264
O	13.308229	-8.880807	-7.513499
O	13.465764	-6.738856	-5.358310
Ti	11.394263	-11.850988	-5.436318
O	9.688638	-12.552319	-5.493360
O	11.326284	-9.611280	-5.471539
O	11.686704	-11.331012	-7.288235
Ti	11.602016	-9.482448	-7.316658
O	11.436382	-7.262069	-7.629282
Ti	9.465153	-4.649135	-5.490182
Ti	9.604590	-6.951836	-7.361635
O	9.489610	-5.161410	-7.401486
O	11.080366	-3.817680	-5.455075
O	9.705017	-9.078825	-7.845211
O	9.502776	-6.772204	-5.459129
Ti	7.985247	-11.845864	-5.555637
O	6.455527	-12.854508	-5.597680
O	7.962202	-9.742477	-5.677921
O	7.987513	-11.438918	-7.510375
Ti	7.884366	-9.615421	-7.629236
O	7.804842	-7.431660	-8.040646
Ti	6.071275	-4.518577	-5.611264
Ti	5.940250	-6.907463	-7.486925
O	5.882994	-5.061849	-7.448776
O	7.785103	-3.856907	-5.551795
O	6.030124	-9.198823	-7.577298
O	6.057511	-6.792766	-5.619832
Ti	4.619169	-12.404211	-5.645067
O	3.539445	-13.930239	-5.816018
O	3.544641	-9.071297	-5.666597
O	4.370680	-11.321359	-7.059458
Ti	4.157181	-9.411271	-7.357606
O	4.286408	-7.484986	-7.989508
O	4.846168	-3.092267	-5.487085
O	2.918653	-9.729956	-8.835231
Ti	13.155274	-6.853600	-3.445082

O	13.027001	-4.951781	-3.628136
O	13.200328	-8.834478	-3.360000
O	11.592009	-11.294532	-3.605036
Ti	11.505664	-9.445647	-3.617003
O	11.305812	-7.218468	-3.107594
Ti	9.469891	-6.901861	-3.553318
O	9.379653	-5.102904	-3.562873
O	9.580234	-9.033677	-3.271195
O	7.848089	-11.301220	-3.678971
Ti	7.787322	-9.500271	-3.813518
O	7.648417	-7.415981	-3.155137
Ti	5.807041	-6.927855	-3.748993
O	5.769927	-5.070860	-3.783148
O	5.906922	-9.185353	-3.679588
O	4.175533	-11.349587	-4.238825
Ti	4.025668	-9.455836	-3.901928
O	4.142950	-7.465775	-3.312347
O	2.672497	-9.559078	-2.567945
O	14.850962	-6.655334	-8.340276
H	15.040165	-5.711007	-8.505070
O	14.433523	-6.623910	-2.043509
H	14.606920	-5.682955	-1.845954
O	14.055928	-2.928431	-5.308288
H	14.233346	-2.640418	-4.392008
H	13.007276	-13.440640	-6.430293
H	4.471722	-2.922427	-4.602229
H	2.575677	-8.923069	-9.265504
H	2.515376	-8.682923	-2.162570
H	3.008210	-14.279738	-6.546776
O	7.296117	-10.276332	-10.121708
O	4.478032	-9.412457	-12.429817
O	3.398123	-11.659119	-10.710947
O	5.676374	-13.604859	-10.359683
O	8.183120	-12.483427	-9.782052
O	7.992665	-7.527180	-10.790019
C	5.092420	-9.953679	-11.205778
C	4.785980	-11.462145	-11.059309
C	6.580066	-9.536841	-11.179904
C	5.771810	-12.163586	-10.119274
C	7.207733	-11.731826	-10.441611
C	6.655034	-8.033729	-10.924138
H	4.583094	-9.438207	-10.392166
H	4.916116	-11.944778	-12.043963
H	7.067915	-9.787207	-12.134520
H	5.539056	-11.931645	-9.073237
H	7.406326	-11.837158	-11.517868
H	6.058271	-7.839966	-10.024487

H 6.167343 -7.530452 -11.763764
H 4.502060 -10.108186 -13.118827
H 3.163049 -11.067226 -9.932967
H 6.427968 -14.000139 -9.865665
H 8.211902 -12.219443 -8.795490
H 8.170958 -7.446421 -9.817761

Cluster structure with a subsurface oxygen vacancy as in figure 5a

67

Coordinates from ORCA-job E= -4125.868699551901 hartree

O	12.334617	-13.463796	-5.853718
Ti	12.798468	-4.305577	-5.660490
Ti	13.186651	-7.354252	-7.366180
O	13.040458	-5.381612	-7.082013
O	12.892941	-9.142432	-8.112716
O	13.804858	-7.719560	-5.644808
Ti	11.044003	-12.095434	-5.681253
O	9.327870	-12.767315	-5.761426
O	11.162025	-9.979967	-5.671169
O	11.269159	-11.562355	-7.572932
Ti	11.243411	-9.722506	-7.545207
O	11.243580	-7.481668	-7.268948
Ti	9.418145	-4.206291	-5.722400
Ti	9.403445	-7.133759	-7.263843
O	9.367280	-5.281864	-7.201849
O	11.084887	-3.449582	-5.686121
O	9.419229	-9.153530	-7.961618
Ti	7.655592	-11.960085	-5.715325
O	6.049653	-12.839677	-5.706134
O	7.602361	-9.857670	-5.659567
O	7.608359	-11.413061	-7.636930
Ti	7.522070	-9.613732	-7.535832
O	7.558100	-7.471620	-7.830789
Ti	6.072064	-3.877930	-5.805834
Ti	5.709766	-6.931925	-7.452108
O	5.845475	-5.006213	-7.205235
O	7.813889	-3.229883	-5.758661
O	5.699083	-9.231568	-7.751096
O	5.612778	-7.314380	-5.685385
Ti	4.220258	-12.356855	-5.680670
O	3.093545	-13.880136	-5.593950
O	3.420902	-9.001905	-5.570761
O	3.848351	-11.245660	-7.083005
Ti	3.801755	-9.358441	-7.345614
O	4.088487	-7.344220	-8.088091
O	4.836172	-2.460522	-6.009949
O	2.312911	-9.275068	-8.532590
Ti	13.131152	-7.372050	-3.959618

O	12.955502	-5.387101	-4.242530
O	12.821784	-9.149514	-3.186930
O	11.209203	-11.598142	-3.814121
Ti	11.198612	-9.742557	-3.791678
O	11.197596	-7.516111	-4.146839
Ti	9.362557	-7.156840	-4.175263
O	9.298771	-5.314885	-4.253126
O	9.398212	-9.152708	-3.319242
O	7.742846	-11.517457	-3.803174
Ti	7.541286	-9.714421	-3.760877
O	7.490651	-7.501807	-3.647826
Ti	5.764669	-6.744551	-3.986998
O	5.765948	-4.870666	-4.274787
O	5.760915	-9.579907	-3.348506
O	3.857974	-11.320530	-4.269499
Ti	3.934626	-9.403896	-3.813991
O	4.291376	-7.432999	-3.228669
O	2.562100	-9.395740	-2.501222
O	14.515986	-6.899657	-8.673720
H	14.683358	-5.935950	-8.682732
O	14.420132	-6.884828	-2.618841
H	14.554420	-5.917645	-2.579188
O	14.181368	-2.984389	-5.529546
H	14.744329	-2.868112	-4.745187
H	12.702535	-13.581269	-6.750626
H	4.090454	-2.432655	-6.632057
H	2.290615	-8.377191	-8.925072
H	2.507338	-8.522699	-2.062788
H	2.421087	-14.051672	-4.914129

Cluster structure with a surface oxygen vacancy as in figure 5b
67
Coordinates from ORCA-job E= -4125.828661857504 hartree

O	13.244783	-13.945689	-3.660541
Ti	15.582429	-5.440547	-4.571320
Ti	15.731478	-7.880808	-6.361557
O	16.350963	-6.064997	-6.164934
O	15.631875	-9.801609	-6.431512
O	15.441242	-7.651558	-4.426512
Ti	12.554463	-12.392052	-4.493986
O	10.964570	-12.737428	-5.346068
O	13.078086	-10.273322	-4.653217
O	13.728957	-12.281056	-6.069614
Ti	13.937165	-10.473853	-6.296600
Ti	12.545548	-4.967295	-6.023695
Ti	13.159746	-7.388638	-7.690712
O	13.348920	-5.573064	-7.683859

O	14.131633	-4.461844	-5.218648
O	12.818306	-9.497468	-7.424661
O	12.126888	-6.986809	-6.024727
Ti	9.645664	-11.602714	-6.045467
O	7.990517	-11.956096	-6.809352
O	10.082955	-9.602776	-5.971658
O	10.575725	-11.267428	-7.821505
Ti	10.822378	-9.505141	-7.743883
O	11.572366	-7.789887	-8.825112
Ti	9.656603	-4.239803	-7.658416
Ti	9.848438	-6.862273	-9.196828
O	10.215445	-4.997085	-9.301919
O	11.262752	-3.882284	-6.802175
O	9.284275	-8.821437	-8.526849
O	9.059037	-6.273448	-7.539475
Ti	6.499669	-11.036555	-7.433174
O	5.110136	-12.264371	-7.858515
O	6.857956	-8.954183	-7.264636
O	6.962498	-10.479412	-9.279968
Ti	7.403669	-8.732989	-9.074336
O	8.534532	-7.312660	-10.318944
O	8.746007	-2.662347	-8.183855
O	5.979614	-7.725609	-9.830621
Ti	14.713659	-7.642931	-2.706196
O	14.915664	-5.812237	-2.745983
O	13.864191	-9.371193	-1.905038
O	12.073663	-11.590660	-2.805966
Ti	12.439955	-9.749205	-2.918705
O	12.826440	-7.669849	-3.351316
Ti	11.352463	-7.016092	-4.289305
O	11.562640	-5.256098	-4.264801
O	10.620981	-8.877015	-3.122959
O	9.016509	-11.087174	-4.311841
Ti	9.158899	-9.260051	-4.252997
O	9.423975	-7.182905	-4.731227
Ti	8.253413	-6.161941	-5.838967
O	8.506451	-4.360584	-5.998396
O	7.326801	-8.894541	-4.140188
O	5.558609	-10.482381	-5.866964
Ti	6.032566	-8.702456	-5.457260
O	6.571611	-6.738904	-5.722102
O	4.349279	-8.031478	-4.862814
O	15.124127	-7.553372	-8.318458
H	15.425000	-6.679000	-8.643000
O	16.125670	-7.834322	-1.392122
H	16.663724	-7.028994	-1.264033
O	16.944949	-4.289087	-3.897920
H	16.771174	-4.019696	-2.973525

H	13.130292	-13.976244	-2.691114
H	8.126734	-2.323483	-7.507913
H	6.277828	-6.912342	-10.284223
H	4.344081	-7.053112	-4.855831
H	4.414228	-12.312901	-7.174622

D-glucose adsorbed on the cluster with a surface vacancy as in figure 6a
91
Coordinates from ORCA-job E= -4812.487533632946 hartree

O	11.964547	-13.633875	-3.062158
Ti	12.622390	-5.148650	-6.237570
Ti	12.843282	-8.145112	-7.552096
O	12.422970	-6.191606	-8.021759
O	12.715260	-9.935483	-6.931796
O	13.149482	-7.192872	-5.818672
Ti	10.874875	-12.335191	-3.885624
O	9.171544	-12.947688	-3.957393
O	10.714767	-10.220918	-4.673722
O	11.416259	-12.428020	-5.731915
Ti	11.176717	-10.723298	-6.414387
Ti	9.320240	-5.411816	-6.674269
Ti	9.631598	-8.166803	-8.103892
O	9.741003	-6.306218	-8.345877
O	10.910575	-4.468971	-6.608405
O	9.652015	-10.152112	-7.316738
O	9.388996	-7.388020	-6.110337
Ti	7.548286	-12.057354	-4.376761
O	5.825871	-12.685258	-4.286742
O	7.554340	-10.036828	-4.861281
O	7.794699	-12.077688	-6.322584
Ti	7.668711	-10.299126	-6.732606
O	7.750881	-8.699526	-8.013389
Ti	5.919949	-5.290534	-7.254494
Ti	5.813347	-8.207142	-8.258065
O	5.871112	-6.324426	-8.831806
O	7.677438	-4.718960	-7.169778
O	5.884797	-10.109531	-7.206055
O	5.691019	-7.286710	-6.549465
Ti	4.081177	-12.016684	-4.409014
O	2.855465	-13.467762	-4.393054
O	4.337479	-9.965952	-4.853437
O	3.643921	-11.876793	-6.395010
Ti	3.934179	-10.126881	-6.709382
O	4.161510	-8.873552	-8.409320
O	4.732333	-3.867153	-7.657084
O	2.170726	-9.409871	-6.783156
Ti	12.839911	-7.094642	-3.922068

O	12.643648	-5.151270	-4.434056
O	12.801223	-8.852126	-3.123852
O	11.083431	-11.104509	-2.349107
Ti	11.024839	-9.395140	-3.031407
O	10.969285	-7.287760	-3.691734
Ti	9.139441	-7.067081	-4.280543
O	9.025114	-5.341416	-4.660627
O	9.170106	-8.683406	-2.679320
O	7.594493	-11.154434	-2.653064
Ti	7.434393	-9.386569	-3.010458
O	7.233829	-7.456052	-3.962369
Ti	5.593335	-6.833528	-4.725833
O	5.491159	-5.105358	-5.290328
O	5.750766	-9.101888	-2.230311
O	3.682432	-11.158662	-2.807493
Ti	4.070205	-9.291137	-2.973856
O	4.168935	-7.466926	-3.848504
O	2.662688	-8.584664	-1.909822
O	13.722762	-8.452750	-9.225206
H	14.285729	-7.842956	-9.742015
O	14.170843	-6.439075	-2.702042
H	14.252483	-5.465851	-2.749889
O	13.997544	-3.913650	-6.621419
H	14.439512	-3.416189	-5.910639
H	12.274786	-13.448333	-2.155998
H	4.387990	-3.399603	-6.871111
H	2.095793	-8.613604	-7.345015
H	2.481124	-7.642333	-2.095051
H	2.501849	-13.681641	-5.278721
O	9.736932	-8.490664	-9.975874
O	8.825213	-7.756034	-13.514496
O	11.353296	-7.004817	-13.816202
O	12.514815	-8.672406	-11.628994
O	14.344389	-6.525080	-11.497687
O	6.452726	-8.160014	-10.434721
C	9.709209	-8.057466	-12.349669
C	10.867431	-7.049281	-12.438172
C	8.911907	-8.002458	-11.025968
C	12.038616	-7.325715	-11.503405
C	13.143537	-6.311276	-11.721044
C	7.586139	-8.799025	-11.117150
H	10.086619	-9.063475	-12.533506
H	10.465918	-6.051725	-12.176182
H	8.665472	-6.938085	-10.832248
H	11.663005	-7.151767	-10.481639
H	12.816151	-5.307487	-12.053174
H	7.726755	-9.763733	-10.629065
H	7.291750	-8.947634	-12.160438

H	8.271127	-6.976867	-13.290022
H	10.568394	-7.209185	-14.373076
H	12.889334	-8.923334	-10.735595
H	11.553246	-5.984271	-8.447941
H	6.482609	-7.167261	-10.333780

D-glucose adsorbed on the cluster with a subsurface vacancy as in figure 6b

91

Coordinates from ORCA-job

O	10.93687473532485	-12.67841439733970	-8.15577332969172
Ti	11.79515956679996	-3.89504191045290	-5.77389946324971
Ti	11.36435899160782	-6.31703459387748	-8.12495175649766
O	11.39618416060297	-4.52707673114674	-7.46342866659793
O	10.53661434682818	-7.68796686013798	-9.28266479988655
O	12.53336434758451	-7.25265437315476	-7.05270328761521
Ti	9.89130796924092	-11.51402748820537	-7.19745929113940
O	8.20975563297476	-12.19137061266551	-6.86366777053085
O	10.00844227010250	-9.48737448974744	-6.60479824176654
O	9.54820995922018	-10.37841454041989	-8.85614893439569
Ti	9.38243894172042	-8.64288498981456	-8.21425109637473
O	9.67316689354064	-6.71554414289377	-7.18317920709695
Ti	8.70392120729141	-4.03373250409435	-4.38007531428261
Ti	7.95570836557571	-6.43872098001057	-6.48742687439939
O	8.04413138438283	-4.67522126125910	-5.96418599789696
O	10.28415297541535	-3.21155149004530	-4.83919178396735
O	7.58795466962964	-8.13515904277797	-7.78392943723935
Ti	6.71656632122265	-11.56827494073462	-5.94851454613827
O	5.20886250072824	-12.52963642725429	-5.56134168154954
O	6.74854797071632	-9.57029279183938	-5.27152519350074
O	5.94454400679946	-10.51383859067477	-7.46192999897349
Ti	5.95497530644527	-8.83025297656492	-6.81853672671447
O	6.02834962810073	-6.71790547871455	-6.40792760052540
Ti	5.58259399486952	-3.93586726992836	-3.10032885650580
Ti	4.46615412259735	-6.43151964145554	-5.25516617889701
O	4.77659796045942	-4.64932088341704	-4.56443236378398
O	7.24467128082049	-3.21374453523845	-3.54225288553287
O	4.21780718413387	-8.50778995258029	-6.22617929742640
O	5.02382853174739	-7.30973126270414	-3.78966981324774
Ti	3.54276134422420	-12.20153797377546	-4.72586325906146
O	2.36871344786147	-13.67043662230973	-4.89397840791558
O	2.95089819958261	-9.08822200763635	-3.33543383265980
O	2.77138290241324	-10.76640914461843	-5.48201146105514
Ti	2.62434167683478	-8.86076759423926	-5.13106090177731
O	2.69099793178833	-6.78455770457041	-5.27969847976749
O	4.44850706537561	-2.60515107538722	-2.40342979920017
O	0.78948518211761	-8.67320476205919	-5.59649177407084
Ti	12.60131687594597	-7.37036832036553	-5.15411746356103

O	12.43846826695081	-5.32227571672173	-4.95918767551722
O	12.56265354894934	-9.27357785865783	-4.93260620493126
O	10.70732680919496	-11.54528241973572	-5.42556934152601
Ti	10.78077538304178	-9.78739631227584	-4.91391863828501
O	10.82114222732799	-7.53160200777149	-4.50440176980850
Ti	9.09926895191075	-7.29283082496260	-3.76557554960785
O	9.11435836678464	-5.49399602613145	-3.33718621048044
O	9.33627722767516	-9.43877184475859	-3.65484809825612
O	7.53776931143785	-11.66256959021167	-4.16864818784308
Ti	7.42211536856369	-9.96957474932485	-3.53092871687675
O	7.54694150928216	-7.88986711692248	-2.74213281704675
Ti	5.83387741304129	-7.21933738132044	-2.17288327922189
O	5.82439010859039	-5.33431792609257	-1.92306071284449
O	5.94334745209401	-10.06947793314600	-2.46341840080677
O	3.75815134842987	-11.64812068389062	-3.00290363866762
Ti	4.07854856878869	-9.97714870965728	-2.11663435519402
O	4.74828961965305	-8.16740605569647	-1.13770862744501
O	3.34988803108730	-10.35839855444027	-0.41255831056129
O	12.44186038519384	-5.74958664809899	-9.68238329127655
H	12.76075409591759	-4.82944596582805	-9.63386954045452
O	14.23948703224245	-7.10737505544982	-4.21629768991868
H	14.39184481093136	-6.16973621307134	-3.98653587953100
O	13.16672058559601	-2.58494781939048	-5.82882410101780
H	14.07668846183076	-2.66063458029315	-5.49937937410699
H	11.33781432435814	-12.46577731208117	-9.03545963633005
H	3.51334559825991	-2.43158869246340	-2.59522707228732
H	0.54434338518462	-7.73011828677694	-5.68195260244103
H	3.53550618411532	-9.62064947511555	0.20422789401427
H	1.47888584413199	-13.68583578346938	-5.28122504109128
O	13.53164444325872	-8.43506940477120	-12.65402708504468
O	11.77901485883802	-11.65230240426332	-13.17094717399343
O	11.36410266852329	-11.33995115292709	-10.49435276129065
O	13.31413150512734	-9.55994548945990	-9.08163912912318
O	14.25569356152221	-7.27129856687473	-10.76398411213040
O	14.05310546090377	-8.52258194237116	-15.26615715124881
C	12.09174363059337	-10.36690261234423	-12.56319850585006
C	12.45398423610507	-10.58359278706152	-11.08725536795667
C	13.25000249568155	-9.71517389542036	-13.29876087573243
C	12.82033839389404	-9.25871795603214	-10.41300196196399
C	13.94038142200432	-8.54406862336187	-11.20750036990559
C	12.97421424377480	-9.35932440596076	-14.75523888212562
H	11.21584044440905	-9.70272758692857	-12.61888815176756
H	13.32504646276216	-11.25168568604989	-11.03795957316503
H	14.13840479359794	-10.36652426301063	-13.23988382343665
H	11.94139740576519	-8.60385591857415	-10.35804791420268
H	14.85464543723292	-9.15581243321168	-11.18543707334726
H	12.01243125045343	-8.82988441394122	-14.82231081281708
H	12.92928166289649	-10.25906523782227	-15.37276117590543

H	11.26891526626164	-12.12856783395018	-12.47373964317251
H	10.65323545802728	-10.80478616184238	-9.99453115934582
H	13.10085588582700	-8.84159003790553	-8.44158177575123
H	13.46739855981122	-6.72086615930365	-10.40827213001180
H	14.26470037953615	-7.91903711874361	-14.51480074685705

[i] A) Perdew, J. P.; Burke, K.; Ernzerhof, M. Generalized Gradient Approximation Made Simple Phys. Rev. Lett. 1996, 77, 3865– 3868; b) Perdew, J. P.; Burke, K.; Ernzerhof, M. Errata: Generalized Gradient Approximation Made Simple Phys. Rev. Lett. 1997, 78, 1396– 1399; c) Adamo, C.; Barone, V. Toward Reliable Density Functional Methods Without Adjustable Parameters: The PBE0Model J. Chem. Phys. 1999, 110, 6158– 6169

[ii] Hongzhi Cheng and Annabella Selloni Surface and subsurface oxygen vacancies in anatase TiO2 and differences with rutile PHYSICAL REVIEW B 79, 092101-4, 2009
